# Reducing the burden of dizziness in middle-aged and older people: A multifactorial, tailored, single-blind randomized controlled trial

**DOI:** 10.1371/journal.pmed.1002620

**Published:** 2018-07-24

**Authors:** Jasmine C. Menant, Americo A. Migliaccio, Daina L. Sturnieks, Cameron Hicks, Joanne Lo, Mayna Ratanapongleka, Jessica Turner, Kim Delbaere, Nickolai Titov, Daniela Meinrath, Catherine McVeigh, Jacqueline C. T. Close, Stephen R. Lord

**Affiliations:** 1 Neuroscience Research Australia, Sydney, New South Wales, Australia; 2 School of Public Health and Community Medicine, University of New South Wales, Sydney, New South Wales, Australia; 3 Graduate School of Biomedical Engineering, University of New South Wales, Sydney, New South Wales, Australia; 4 School of Medical Sciences, University of New South Wales, Sydney, New South Wales, Australia; 5 Department of Psychology, Macquarie University, Sydney, New South Wales, Australia; 6 Department of Physiotherapy, Prince of Wales Hospital, Sydney, New South Wales, Australia; 7 Prince of Wales Clinical School, University of New South Wales, Sydney, New South Wales, Australia; Massachusetts General Hospital, UNITED STATES

## Abstract

**Background:**

Dizziness is common among older people and is associated with a cascade of debilitating symptoms, such as reduced quality of life, depression, and falls. The multifactorial aetiology of dizziness is a major barrier to establishing a clear diagnosis and offering effective therapeutic interventions. Only a few multidisciplinary interventions of dizziness have been conducted to date, all of a pilot nature and none tailoring the intervention to the specific causes of dizziness. Here, we aimed to test the hypothesis that a multidisciplinary dizziness assessment followed by a tailored multifaceted intervention would reduce dizziness handicap and self-reported dizziness as well as enhance balance and gait in people aged 50 years and over with dizziness symptoms.

**Methods and findings:**

We conducted a 6-month, single-blind, parallel-group randomized controlled trial in community-living people aged 50 years and over who reported dizziness in the past year. We excluded individuals currently receiving treatment for their dizziness, those with degenerative neurological conditions including cognitive impairment, those unable to walk 20 meters, and those identified at baseline assessment with conditions that required urgent treatment. Our team of geriatrician, vestibular neuroscientist, psychologist, exercise physiologist, study coordinator, and baseline assessor held case conferences fortnightly to discuss and recommend appropriate therapy (or therapies) for each participant, based on their multidisciplinary baseline assessments. A total of 305 men and women aged 50 to 92 years (mean [SD] age: 67.8 [8.3] years; 62% women) were randomly assigned to either usual care (control; *n* = 151) or to a tailored, multifaceted intervention (*n* = 154) comprising one or more of the following: a physiotherapist-led vestibular rehabilitation programme (35% [*n* = 54]), an 8-week internet-based cognitive-behavioural therapy (CBT) (19% [*n* = 29]), a 6-month Otago home-based exercise programme (24% [*n* = 37]), and/or medical management (40% [*n* = 62]). We were unable to identify a cause of dizziness in 71 participants (23% of total sample). Primary outcome measures comprised dizziness burden measured with the Dizziness Handicap Inventory (DHI) score, frequency of dizziness episodes recorded with monthly calendars over the 6-month follow-up, choice-stepping reaction time (CSRT), and gait variability. Data from 274 participants (90%; 137 per group) were included in the intention-to-treat analysis. At trial completion, the DHI scores in the intervention group (pre and post mean [SD]: 25.9 [19.2] and 20.4 [17.7], respectively) were significantly reduced compared with the control group (pre and post mean [SD]: 23.0 [15.8] and 21.8 [16.4]), when controlling for baseline scores (mean [95% CI] difference between groups [baseline adjusted]: −3.7 [−6.2 to −1.2]; *p* = 0.003). There were no significant between-group differences in dizziness episodes (relative risk [RR] [95% CI]: 0.87 [0.65 to 1.17]; *p* = 0.360), CSRT performance (mean [95% CI] difference between groups [baseline adjusted]: −15 [−40 to 10]; *p* = 0.246), and step-time variability during gait (mean [95% CI] difference between groups [baseline adjusted]: −0.001 [−0.002 to 0.001]; *p* = 0.497). No serious intervention-related adverse events occurred. Study limitations included the low initial dizziness severity of the participants and the only fair uptake of the falls clinic (medical management) and the CBT interventions.

**Conclusions:**

A multifactorial tailored approach for treating dizziness was effective in reducing dizziness handicap in community-living people aged 50 years and older. No difference was seen on the other primary outcomes. Our findings therefore support the implementation of individualized, multifaceted evidence-based therapies to reduce self-perceived disability associated with dizziness in middle-aged and older people.

**Trial registration:**

Australian New Zealand Clinical Trials Registry ACTRN12612000379819.

## Abstract 2

In a multifactorial randomized controlled trial Jasmine C. Menant and colleagues examined whether a multidisciplinary approach with individually, cause specific interventions would improve dizziness in middle-aged and older people.

## Author summary

**Why was this study done?:**

- Dizziness is a frequent complaint amongst middle-aged and older people.
- Dizziness can lead to poor health outcomes, including reduced quality of life, depression, and falls.
- The increasingly multifactorial nature of dizziness with advancing age makes it difficult to objectively establish a diagnosis and offer effective interventions.
- Although few multidisciplinary studies have been conducted, none to date have examined whether tailoring the intervention to the specific cause(s) of dizziness can improve dizziness in middle-aged and older community-dwellers.

**What did the researchers do and find?:**

- We assessed 305 community-dwellers aged 50 years and above who reported dizziness in the past year on a range of questionnaires and tests of sensory and physical function, mental health, quality of life, and cardiovascular function, at baseline and 6 months.
- We randomly assigned the participants to usual care (control; *n* = 151) or to a 6-month tailored, multifaceted intervention (*n* = 154) comprising one or more of the following: vestibular rehabilitation (35% [*n* = 54]), cognitive-behavioural therapy (CBT) for anxiety and depression (19% [*n* = 29]), a home-based exercise programme to train balance and lower-limb strength (24% [*n* = 37]), and/or medical management (40% [*n* = 62]).
- We found that the multifactorial tailored intervention was effective in improving dizziness-related quality of life but did not affect balance, gait, or the frequency of dizziness episodes over 6 months.

**What do these findings mean?:**

- Our pragmatic study findings suggest that prescribing middle-aged and older people with dizziness evidence-based therapies directly targeting their deficits is likely to reduce their dizziness handicap but might not improve their physical function.
- Low-dizziness handicap on entry to the study and the inclusion of participants not currently receiving a dizziness treatment might partly explain the lack of intervention effect on balance and gait, as well as explain why we were unable to identify a cause of dizziness in 71 participants (23% of total sample).
- We suggest that community-based dizziness clinics use existing healthcare services to implement tailored and multifaceted dizziness interventions based on multidisciplinary assessments.

## Introduction

Dizziness is common in older people: prevalence rates in the community range between 10% and 30% [1–4] and increase with age [1,2,5]. Dizziness is often associated with a marked increase in self-reported functional disability [1,6], depressive symptoms [3,4], decreased participation in social activities, poor self-reported health, and reduced falls efficacy [4]. In addition, the risk of experiencing multiple falls is significantly heightened among older people who report dizziness in the past [7], and this is likely to translate into an increased number of fall-related injuries. In fact, cross-sectional analysis from the 2008 United States National Health Interview Survey reveals that, among people who had reported a fall in the past 12 months, those who had reported dizziness or balance problems in that time were at 1.5-fold–increased odds of injury from the fall compared with their healthy peers (46% versus 36%) [8], and this association remained significant after controlling for age and sex [9]. Dizziness is a subjective complaint that is commonly referred to by patients as vertigo, light-headedness, imbalance, or a ‘floating sensation’; terms that have traditionally been associated with vestibular, cardiovascular, balance, and psychological disorders, respectively. An additional complication is the variation in the natural course of dizziness that is very dependent on etiology, i.e., differential patterns in the progression from mild to severe forms, the addition of secondary symptoms such as anxiety, and potential spontaneous regression.

There is good evidence from Cochrane reviews, systematic reviews, and large randomized controlled trials for effective therapies addressing aspects of each disorder: vestibular rehabilitation for peripheral vestibular disorders (including repositioning manoeuvres for benign paroxysmal positional vertigo [BPPV]) [10], syncope assessment and management [11,12], balance and strength training for older people [13,14], and cognitive-behavioural therapy (CBT) for patients with psychogenic dizziness [15]. Despite the availability of such effective treatments, a significant number of cases of dizziness remain unresolved—20% to 45%—because the cause of dizziness could not be identified [16,17].

Older people often have symptomatology suggestive of more than one subtype of dizziness [18–21]. Yet multidisciplinary interventions addressing this symptom have been scarce. Most published trials to date have focused on a single therapy: vestibular rehabilitation (e.g., [22–24]), balance training [25], CBT [26], or pharmacological therapy [27]. To our knowledge, only a few trials targeting vertigo/dizziness published to date were multidisciplinary, combining vestibular rehabilitation and CBT [28–30]; balance training and vestibular rehabilitation [31]; and education, vestibular rehabilitation, and CBT [24,32]. Three of these studies [28,30,32], including a large randomized controlled trial [28], pointed to significant improvement in dizziness handicap post intervention. Yet they either involved a highly selected sample of patients (e.g., primary care patients with labyrinthine deficits [28])—precluding a potential tailoring of the intervention—or were of a pilot nature, involving short follow-ups and fewer than 45 participants [30,32].

In summary, despite significant evidence of the multifactorial nature of dizziness with advancing age and the availability of a range of evidence-based therapies addressing multiple aspects of dizziness, no study to date has investigated the effectiveness of a multidisciplinary intervention to improve dizziness in the general older population. Therefore, within a randomized controlled trial design, we designed a comprehensive, multidisciplinary battery of vestibular, cardiovascular, neuromuscular, balance, and psychological assessments to improve the likelihood of obtaining a diagnosis for the symptom of dizziness in middle-aged and older people. The primary aim was to compare the effect of a tailored multifaceted intervention versus usual care on frequency of dizziness episodes, dizziness handicap, balance, and gait in people aged 50 years and over with self-reported dizziness. Secondary aims were to determine the effects of the programme on physiological falls risk, cardiovascular, and psychological measures associated with dizziness.

## Methods

### Study design

We conducted a 6-month single-blind, parallel-group randomized controlled trial in a single research centre (Neuroscience Research Australia, NeuRA) in Sydney, Australia. The trial was approved by the Human Research Ethics Committee of the University of New South Wales (HC 12152). All eligible participants provided written informed consent prior to participating in the study. Details of the protocol and study design have been reported previously [33] and can be found in the online supplemental material (S2 Text) and on the following webpage: https://bmcgeriatr.biomedcentral.com/articles/10.1186/s12877-017-0450-3. The study protocol is registered in the Australia New Zealand Clinical Trials Registry ACTRN12612000379819. A data safety monitoring committee involving a NeuRA senior scientist not involved in the study and the resident biostatistician oversaw the study.

### Participants

We recruited community-living middle-aged and older adults through advertisements; flyers in community facility, hospital, and university noticeboards; articles in newspapers and newsletters for older people; the NeuRA website, newsletter, and mailing list; and by mailbox drops within the local community. After an initial phone screening, eligible individuals were invited to participate if they (i) were aged 50 years and over, (ii) had experienced at least one significant episode of dizziness in the past 12 months, (iii) lived independently in the community, and (iv) were able to understand the English language. Individuals were excluded if they (i) had a degenerative neurological condition, (ii) were currently receiving treatment for their dizziness, (iii) had a cognitive impairment (General Practitioner assessment of Cognition [GPCOG] score <5) [34], and/or (iv) were unable to walk 20 metres without difficulty, with or without the use of a walking aid. Participants identified at baseline assessment with conditions that required urgent treatment such as suspected stroke, transient ischemic attack, or other undiagnosed neurological or acute cardiovascular condition, severe depressive, or severe anxiety symptoms (Patient Health Questionnaire nine-item [PHQ-9] [35]: total score ≥20; or Generalized Anxiety Disorder seven-item [GAD-7] [36]: total score of ≥20, respectively; both with or without expression of suicidal thoughts) were also excluded from the study and were referred following consent for appropriate treatment.

### Randomization and masking

Participants were randomly assigned to the intervention or control groups following baseline assessment and case conference, using a computer-generated random number schedule with permuted blocks (sizes: 2, 4, and 6). Allocation was concealed by using central randomization performed by NeuRA personnel not otherwise involved in the study. Outcome assessors, including those monitoring the dizziness episodes, were masked to study group allocation. Due to the nature of the intervention, it was not possible to blind the staff administering interventions or the participants. Participants were instructed not to inform the assessors of their intervention status.

### Procedures

All eligible participants attended NeuRA for a 3-hour baseline assessment undertaken by trained research staff. The assessments included diagnostic tests for descriptive purposes (e.g., clinical tests of vestibular function) and for allocating intervention participants to treatment arms as well as baseline measures for the primary and secondary outcomes. Participants returned to NeuRA for reassessment of outcomes at the end of the trial (6 months post randomization).

Multidisciplinary case conferences were held fortnightly throughout recruitment. At these meetings, consensus recommendations and baseline assessment results were used to prioritize and guide appropriate therapies for each participant [33]. Panel members included a geriatrician, a vestibular neuroscientist, a psychologist, an exercise physiologist, the study coordinator, and a baseline assessor. The intervention plan was guided by published normative data for the sensorimotor, balance, and psychological tests as well as the presence of abnormal results in our vestibular and cardiovascular tests. Due to the multifactorial aetiology of dizziness in older people, many participants required multiple interventions that were implemented in a staged manner within the 6-month follow-up period. The therapies selected to address the different aspects of dizziness—vestibular rehabilitation, Otago home-based exercise programme, CBT, and medical management—have been shown to be effective in addressing aspects of each disorder [10–15], as it was our intention to include evidence-based therapies available within health services in our multifaceted intervention. The interventions are outlined below.

#### Vestibular rehabilitation

For participants with vestibular disorders, vestibular rehabilitation was administered at the study site, during a single or several sessions depending on the pathology and its progress, by a physiotherapist (DM) specialized in vestibular rehabilitation. For patients presenting with BPPV, the physiotherapist performed canalith repositioning manoeuvres to treat the different variances of BPPV by removing vestibular debris from the semicircular canals [13]. Given the high prevalence of posterior canal BPPV, the Epley manoeuvre was the most commonly used. In most cases, BPPV symptoms would resolve after one treatment. However, if the patient was still symptomatic after the manoeuvre, the therapist performed a repeat manoeuvre at the next available opportunity, preferably within the next 24 hours. Other peripheral vestibular conditions were managed over 2 to 4 weeks with home-based exercises prescribed according to evidence-based best practice [13] and principles of adaptation, substitution, and habituation [37]. Patients with unilateral peripheral vestibulopathy were prescribed a combination of gaze stabilization exercises (e.g., Cawthorne-Cooksey exercises to stabilize the vestibulo-ocular reflex) as well as static and dynamic balance exercises (e.g., reducing the base of support, altering the support surface while standing and walking). Patients with bilateral peripheral vestibulopathy were prescribed substitution exercises to potentiate the use of alternate inputs to the vestibular system (e.g., potentiating the neck reflexes by asking participants to equate neck movement with head and eye movement when looking between targets on the right and left of midline) and habituation exercises (e.g., encouraging the patient to repeat activities that moderately provoke their symptoms to desensitize them), along with a combination of static and dynamic balance exercises.

In both instances, during an initial 45-minute-long consultation, the physiotherapist reviewed the patients history, assessed them, and then prescribed 2 to 5 exercises (5- to 10-minute sets) for the patients to practice, a minimum of twice and up to 4 times a day, until symptom resolution. Subsequent appointments were scheduled 2 weeks apart to review the participants’ symptomatology and performance of the exercises and modify and/or increase the exercises and their intensity accordingly. An example of exercise progression would involve the transition from practicing static gaze stabilization exercises (during sitting or standing) to dynamic ones (while walking). All cases were resolved within 2 to 4 weeks, which is after 1 to 3 visits to the vestibular physiotherapist.

#### Home-based Otago exercise programme

The home-based Otago exercise programme was delivered by an exercise physiologist for participants with lower-limb muscle weakness and poor balance [13]. This evidence-based fall-prevention programme involved individualized prescription of strength and balance training over 6 home visits. Home visits were scheduled at weeks 0, 1, 2, 4, 8, and 16, and a phone call was scheduled at 12 weeks. During the home visits, the exercise physiologist reviewed exercise adherence, prescribed new exercises, and/or increased the exercises’ intensity by increasing the number of sets, repetitions, and/or asking participants to wear ankle cuff weights. Participants received a booklet with instructions for each exercise prescribed as well as a set of ankle cuff weights (0.5 kg to 4 kg). Typical lower-limb strengthening exercises included calf raises, stair walking, and knee bends. Typical balance exercises included walking sideways and heel-to-toe standing. Participants were required to fill in weekly diaries detailing the number of repetitions achieved for each prescribed exercise and the day on which it was practiced. Self-reported adherence was rated as participants performing 25%, 50%, 75%, 100%, or 100%+ of the prescribed exercises in a given week. Participants were encouraged to exercise a minimum of 3 times per week (approximately 30 minutes each time, recommended 2 hours per week) for 6 months. Details of the publicly available programme can be found online (https://www.acc.co.nz/assets/injury-prevention/acc1162-otago-exercise-manual.pdf). The timing of the last home visit was brought forward to 16 weeks to align within the 6-month intervention.

#### CBT

Participants with anxiety, depression, and/or fear of falling were provided with an online or booklet-based CBT programme with regular telephone support from a registered psychologist [38,39]. The CBT programme involved 5 sessions, including a weekly homework assignment over 8 weeks. The Wellbeing and the Wellbeing Plus courses for people aged up to 59 years old and for those aged 60 years and other, respectively, are internet-based treatment courses designed to teach people about anxiety and depression as well as how to manage their symptoms. These interventions teach core transdiagnostic psychological principles and skills—such as cognitive challenge, exposure, and behavioural activation—that have been found to be effective at reducing symptoms of depression and anxiety in older adults [38,39]. The course consists of 5 online lessons, homework assignments, and case-enhanced stories that detail the experiences of adults (older adults in Wellbeing Plus programme) recovering from symptoms of depression and anxiety. Each lesson is presented in a slideshow format that combines didactic material (i.e., text-based instructions and information) with case-enhanced stories. Participants are instructed to read the lessons over 8 weeks according to a timetable. Participants also receive regular automatic emails that notify them of new course materials as well as reinforce completion of materials and practice of skills. Participants are encouraged to complete 1 lesson every 7 to 10 days and to attempt to regularly practice the skills covered within the lesson summaries. Participants also receive weekly contact from clinicians during treatment. Two registered and experienced psychologists provided all clinical contact with participants, via telephone or a secure email system. In accordance with previous research [40], clinicians were instructed to (a) answer participants’ questions, (b) summarise content, (c) encourage skills practice, (d) resolve difficulties applying skills, and (e) reinforce progress. Clinicians were instructed not to introduce therapeutic skills not covered within the course and, unless clinically indicated, to limit the time spent in contact with or contacting participants to approximately 10 to 15 minutes per week.

#### Medical management

Medical management intervention was divided into complex and simple cases. For complex cases, such as those with multiple comorbidities, frailty, cardiovascular problems (including orthostatic hypotension and abnormal electrocardiogram findings), high physiological risk of falls and/or history of multiple falls, multiple medication use and likely drug interactions, etc.—we organised an appointment to an outpatient Falls Clinic staffed by a geriatrician and a physiotherapist. Individualised interventions following comprehensive geriatric assessment comprised medical and medication management by a geriatrician and strength and balance training. For simpler issues, we sent a letter to participants’ general practitioners outlining factors identified at the baseline assessment that could contribute to dizziness or imbalance. These included problematic medication use, an abnormal electrocardiogram finding, the presence of low blood pressure that could be addressed with fluid intake or medication management, etc., undiagnosed reduced lower-limb sensation that warranted follow-up, and the use of multifocal glasses that could increase fall risk during ambulatory outdoor activities.

#### Control group

During the 6-month trial period, participants assigned to the control group received usual care, i.e., care as usually received by patients in daily practice in this pragmatic clinical trial [41]. We did not give any specific instructions to the control participants. At completion of the trial, participants were provided with their baseline and reassessment reports and offered referrals for recommended interventions.

### Outcomes

The primary outcome measures captured 4 crucial aspects of the trial: dizziness episodes, dizziness handicap, balance, and walking stability. The total number of dizziness episodes during the 6-month follow-up were monitored with monthly dizziness diaries (S3 Text) and follow-up telephone calls as required. The Dizziness Handicap Inventory (DHI) score was computed from a 25-item scale that assesses an individual’s perception of handicap due to dizziness and encompasses emotional, functional, and physical burden [42]. Increased DHI scores have been significantly associated with low mental and physical health-related quality of life, as well as with increased emotional distress [43]. Scores of 0 to 30 represent mild symptoms, 31 to 60 moderate symptoms, and 61 to 100 severe symptoms [42]. Balance was assessed using the choice-stepping reaction time (CSRT), a validated measure of fall risk that also incorporates strength and reaction time [44]. Based on published data, normal mean times for CSRT should range between 750 to 1,200 milliseconds [44]. Walking stability was assessed by the mean step-time variability (coefficient of variation of step time; seconds), recorded as participants performed 3 walking trials at self-selected speed along on a 4-m-long electronic mat placed in the middle of an 8-m-long walkway [45].

Secondary outcome measures were used to elucidate how the interventions might have assisted in ameliorating dizziness symptoms. These measures included assessments of the following: orthostatic hypotension (positive tilt table test) [46], composite physiological fall risk (physiological profile assessment) [47], dynamic balance (coordinated stability) [48], fear of falling (Iconographical Falls Efficacy Scale) [49], anxiety (GAD-7 scale; GAD-7 score >7 indicates clinically significant anxiety symptoms) [36], depression (PHQ-9 scale; PHQ-9 score >9 indicates clinically significant depressive symptoms) [35], and neuroticism (scale from the NEO Five-Factor Inventory) [50]. Clinical tests of vestibular function including the Dix-Hallpike test of BPPV [10] and the head-shake test for the presence of vestibular asymmetry [51] were also conducted. These vestibular tests were not secondary outcome measures (according to our clinical trial registration and published protocol) but were used for diagnostic purposes and for allocating participants into treatment arms. Therefore, we have not presented the results of these tests in the Results section.

Adverse events (e.g., a fall during an exercise session) were monitored with monthly calendars and telephone calls as required. Uptake and adherence to all interventions was documented with therapist records and/or participant diaries. Deviations from the original protocol (clinical registration) were the inclusion of participants aged 50 to 64 years to increase generalizability to a wider demographic, as well as removal of the vertigo symptom scale as a secondary outcome measure because it was clear early in the study that this scale was not useful in documenting types of dizziness and was poorly completed by participants. Cost-effectiveness of the intervention will be addressed in a subsequent paper.

### Statistical analysis

A power analysis determined that 300 participants (150 per group) were required to provide 80% power to detect a statistically significant 20% between-group difference in the primary outcome measures. For these calculations, we assumed an alpha of 0.05 and a dropout rate of 15%. We assumed the following control group means (SDs) based on values from previous studies of older people: CSRT: 1,322 (331) milliseconds; step-time variability: 0.02 (0.01) seconds; and DHI score: 37 (2) [44,45,52,53]. These data represent clinically significant differences based on relevant randomized controlled trials and cohort studies [22,23,28,52,54,55].

All analyses were completed with an intention-to-treat approach (we analysed all available data in the groups to which participants were allocated), and analysis of the primary outcomes was conducted masked to group allocation (S4 Text). Due to the Poisson-like distribution of the dizziness episodes, these data were contrasted between groups with negative binomial regression adjusting for length of follow-up and the outcome presented as a relative risk (RR) (95% CI). Non-normally distributed continuous variables were presented as median (interquartile range) in addition to mean (SD). Between-group comparisons of retest performance for the continuously scored primary and secondary outcome measures were made using generalized linear models controlling for baseline performance. A priori–specified analyses focusing on participants eligible for the vestibular rehabilitation, exercise, CBT, and/or medical management interventions were also conducted for relevant outcome measures. Multiple imputations of the missing primary outcome data were conducted followed by sensitivity analyses; the results are provided in the supplemental files (S5 Text). Values of *p* < 0.05 were considered statistically significant. Holm-Bonferroni adjustments for multiple comparisons were also performed for the primary outcome measures. Analyses were conducted using the SPSS Version 24.0 software package (IBM, Armonk, NY).

## Results

**Fig 1:**
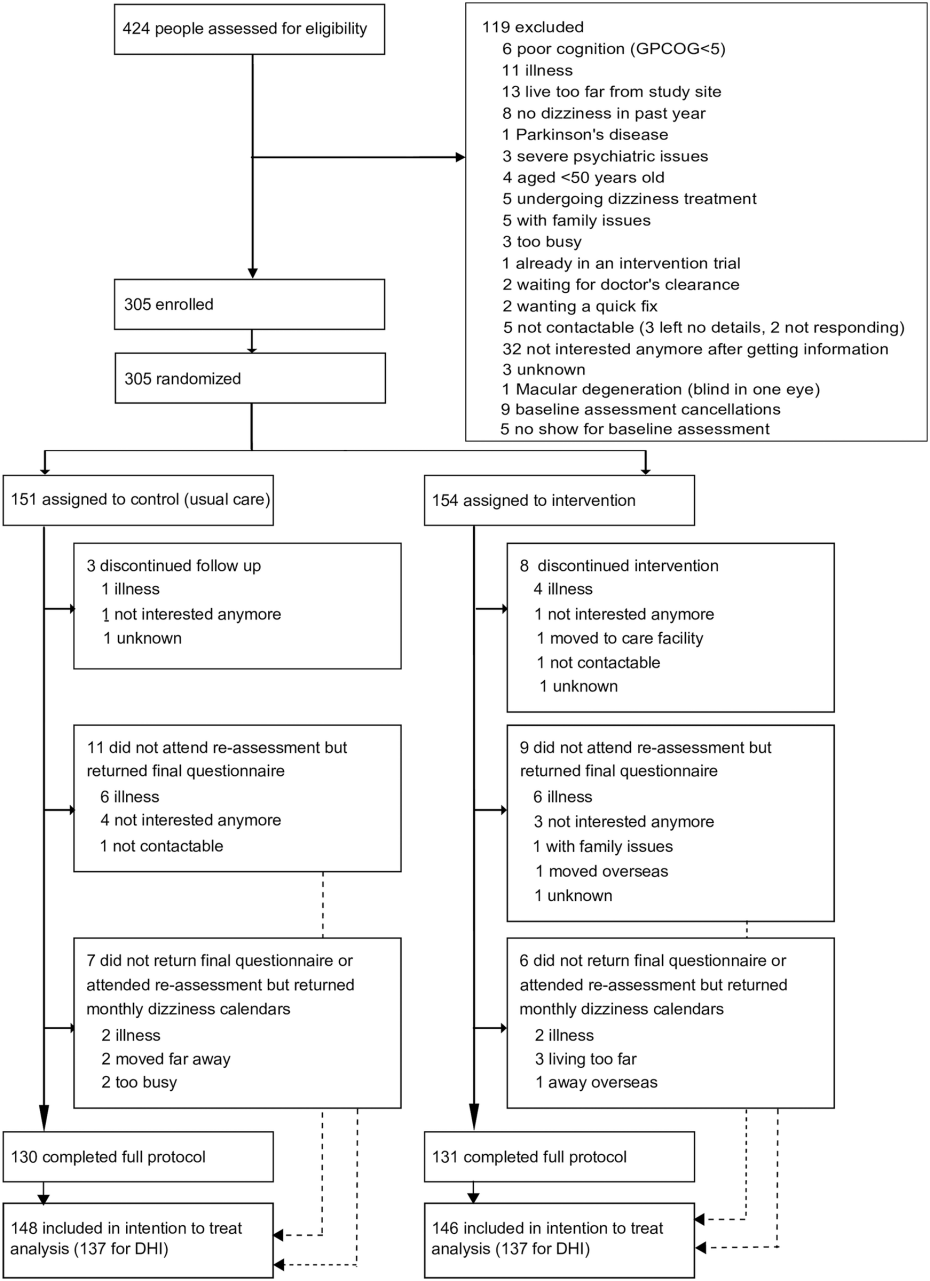
CONSORT diagram (CONSORT; GPCOG; DHI). CONSORT, Consolidated Standards of Reporting Trials; DHI, Dizziness Handicap Inventory; GPCOG, General Practitioner assessment of Cognition.

A total of 424 individuals were assessed for eligibility to participate in the study. Predominantly because they did not satisfy the inclusion criteria or declined to participate (Fig 1), 119 participants were deemed ineligible. Between 15 October 2012 and 2 March 2015, 305 people were recruited, consented to participate, and were then randomly allocated to the control (*n* = 151) or intervention (*n* = 154) groups. The included participants (62% women; *n* = 190) were aged 50 to 92 years (mean [SD]: 67.8 [8.3]). All had intact cognitive function according to their performance on the GPCOG tool of cognitive impairment. Participants in the 2 groups were well-matched with regard to baseline characteristics (Table 1).

**Table 1:** Baseline characteristics of the intention-to-treat population.

	Control (*n* = 151)	Intervention (*n* = 154)
Age, mean (SD), years	67.6 (8.0)	68.0 (8.6)
No. (%) women	101 (67)	92 (60)
GPCOG, mean (SD), score (out of 9)	8.6 (0.7)	8.5 (0.8)
Symptom duration, months, mean (SD)	116 (163)	128 (176)
Symptom duration, months, median (IQR)	42 (18–120)	57 (14–147)
No. (%) with ≥2 falls in previous year	22 (15)	18 (12)
No. (%) with hearing impairment	50 (33)	53 (34)
No. (%) with vision impairment	37 (25)	37 (24)
No. (%) with diagnosed depression	22 (15)	13 (8)
No. (%) with diagnosed anxiety	15 (10)	12 (8)
No. (%) with history of head injury with loss of consciousness	21 (14)	21 (14)
No. (%) with self-reported neck pain	70 (46)	78 (51)
No. (%) with unexplained collapses	14 (9)	19 (12)
Prescription medications, mean (SD), number	3.3 (3.1)	2.9 (2.5)
No. (%) with cardiovascular medications	83 (55)	79 (51)
No. (%) with medications acting on the nervous system	60 (40)	48 (31)
DHI, mean (SD), score	23.6 (16.7)	27.1 (19.5)
No. (%) DHI		
Mild handicap (0–30)	110 (73)	100 (65)
Moderate handicap (31–60)	38 (25)	43 (28)
Severe handicap (61–100)	3 (2)	11 (7)
CSRT, mean (SD), milliseconds	1,059 (175)	1,078 (203)
Step-time variability, mean (SD), s	0.014 (0.008)	0.015 (0.009)

Abbreviations: CSRT, Choice-Stepping Reaction Time; DHI, Dizziness Handicap Inventory; GPCOG, General Practitioner assessment of Cognition score; IQR, interquartile range.

Baseline assessments combined with dizziness history identified at least one potential cause of dizziness for most participants (77%; *n* = 234 of total *n* = 305: 1 cause, *n* = 115 [38%]; 2 causes, *n* = 92 [30%]; 3 to 4 causes, *n* = 27 [9%]). A total of 126 participants (41%) were identified with vestibular disorders (suspected BPPV [*n* = 71], other peripheral vestibular problem [*n* = 48], vestibular migraines [*n* = 7]), 54 (18%) with clinically significant symptoms of anxiety and/or depression, 79 (26%) with lower-limb weakness and poor balance, and 18 (6%) with multiple comorbidities. In addition, 107 participants (35%) had medical or medication issues that could precipitate dizziness warranting treatment or advice from a general practitioner. In 71 participants (23%), no cause of dizziness could be identified from the assessments, questionnaires, and interviews.

Two participants fainted at baseline assessment (S1 Table) and were medically managed appropriately. No serious adverse events were reported over the course of the multifaceted intervention.

**Fig 2:**
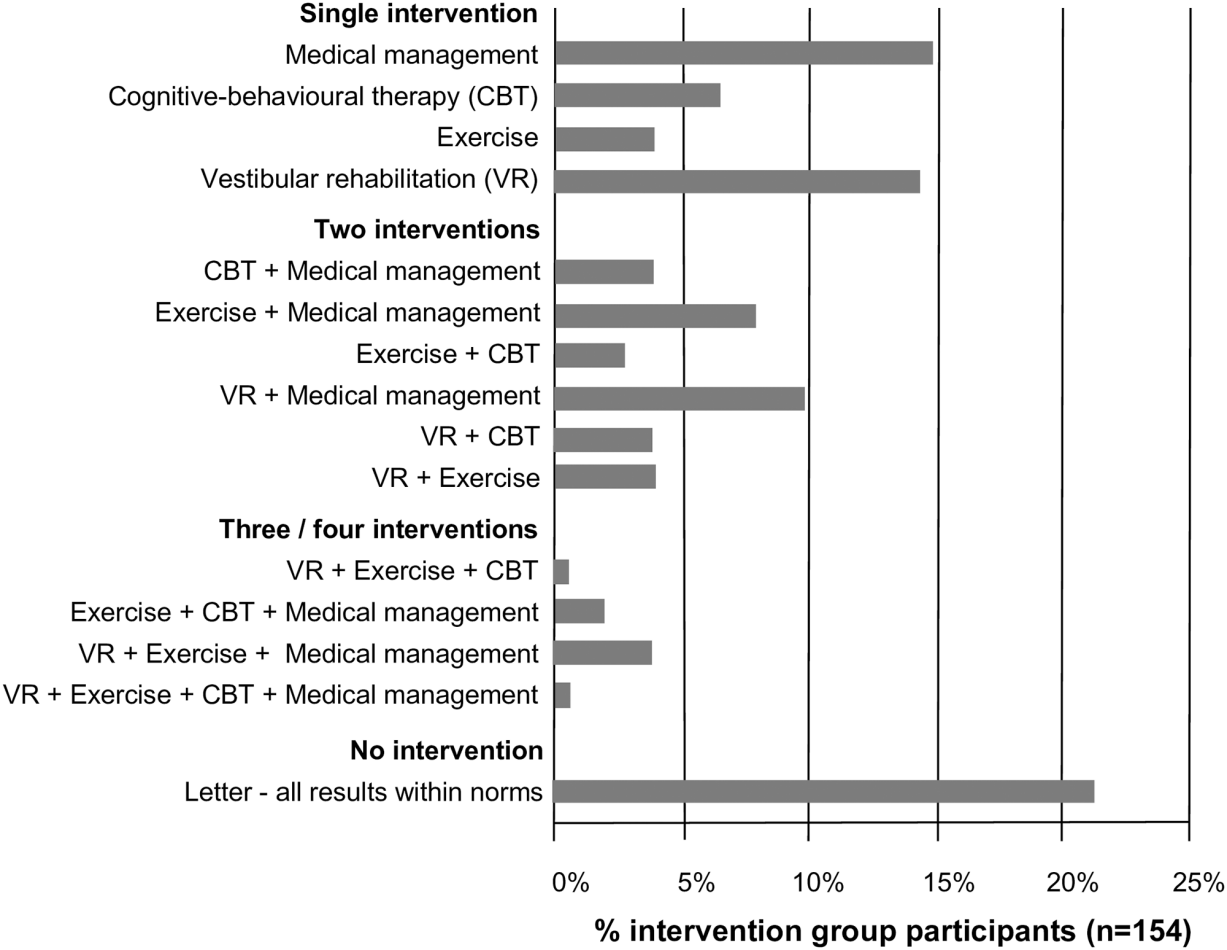
Percentage of intervention group participants (*n* = 154) assigned to the range of intervention combinations (CBT; VR). CBT, cognitive-behavioural therapy; VR, vestibular rehabilitation.

For the 154 participants randomly assigned to the intervention group, 21% were assigned no intervention, 39% assigned a single intervention, 32% assigned 2 interventions, and 8% assigned 3 or 4 interventions (Fig 2).

### Intervention uptake and adherence

There was a 79% (*n* = 45) uptake into the vestibular rehabilitation for those assigned to that therapy (*n* = 57)—5 participants could not be contacted by the vestibular physiotherapist, 2 declined participation because they lived too far from the assessment site, 2 others declined participation because they were not feeling dizzy anymore, 2 had medical issues, and 1 was not interested anymore. All of those who took up the vestibular rehabilitation programme did attend all their sessions with the vestibular physiotherapist. Uptake for the Otago home exercise programme in those assigned to that intervention (*n* = 39) was 85% (*n* = 33)—4 participants discontinued the programme for medical reasons, 1 was erroneously not enrolled in the programme, and 1 moved to a care facility. Regarding adherence to the Otago home exercise programme, 25 of 33 assigned participants (76%) completed 75% or more of the recommended number of exercise sessions (32 of 33 completed 50% or more of the recommended number of sessions). Uptake for the CBT programme was 31% among intervention participants whose anxiety and depression scores met the criteria for inclusion in this therapy (*n* = 31)—6 declined (1 was too busy, 1 had medical reasons, 1 had just moved to a care facility, 2 were no longer interested, and 1 was unknown), 1 could not be contacted, and 15 expressed interest and enrolled in the course but never completed the modules. All the participants who took up the CBT completed the programme. Finally, among participants requiring medical management, only 2 of 9 intervention participants eligible for a falls clinic appointment took up the recommendation. Reasons for failing to attend the falls clinic intervention included living out of area and declining to attend (1), failing to obtain a referral from their general practitioner (4), concurrent referral by general practitioner to a neurology clinic (1), or already attending a community-health programme (1).

### Effect of the multifaceted tailored intervention on primary outcomes

At the completion of the 6-month trial and when controlling for baseline performance, DHI scores in the intervention group were significantly reduced compared with the control group (mean [95% CI] difference between groups [baseline adjusted]: −3.7 [−6.2 to −1.2]; *p* = 0.003). There were no statistically significant between-group differences in dizziness episode frequency during the follow-up period, when controlling for length of follow-up (RR [95% CI]: 0.87 [0.65 to 1.17]; *p* = 0.360). There were also no statistically significant between-group differences in CSRT performance (mean [95% CI] difference between groups [baseline adjusted]: −15 [−40 to 10]; *p* = 0.246) and step-time variability during gait (mean [95% CI] difference between groups [baseline adjusted]: −0.001 [−0.002 to 0.001]; *p* = 0.497) (Table 2).

**Table 2:** Primary outcome measures for the intervention and control groups at baseline and follow-up assessments.

	Baseline			Follow-up		Mean (95% CI) difference between groups^a^ or RR (95% CI), *p*^b^, *p*_adj_^c^, ES_adj_^d^
	Control (*n* = 148)		Intervention (*n* = 146)	Control (*n* = 137)	Intervention (*n* = 137)	
**DHI, mean (SD), score**	23.0 (15.8)		25.9 (19.2)	21.8 (16.4)	20.4 (17.7)	**−3.7 (−6.2 to −1.2), *p* = 0.003, *p***_**adj**_ **= 0.012, ES**_**adj**_ **= 0.25 (0.08 –[−0.17])**
**Dizziness frequency (total number over 6 months), median (IQR)**				33 (10 to 120)	37 (10 to 106)	RR = 0.87 (0.65 to 1.17), *p* = 0.360, *p*_adj_ = 0.720, ES_adj_ = N/A
**Follow-up length, median (IQR), days**				197 (185 to 219)	202 (187 to 228)	N/A
**CSRT, median (IQR), mean (SD), milliseconds**	1,022 (934 to 1,148); 1,056 (175)	1,034 (951 to 1138); 1,066 (194)		1,011 (929 to 1,119); 1,032 (148)	1,009 (932 to 1,092); 1,027 (171)	−15 (−40 to 10), *p* = 0.246, *p*_adj_ = 0.738, ES_adj_ = 0.08 (0.03 –[−0.05])
**Step-time variability, median (IQR), mean (SD), s**	0.012 (0.010 to 0.017); 0.014 (0.008)	0.013 (0.009 to 0.017); 0.015 (0.009)		0.013 (0.009 to 0.017); 0.014 (0.008)	0.012 (0.009 to 0.016); 0.015 (0.008)	−0.001 (−0.002 to 0.001), *p* = 0.497, *p*_adj_ = 0.497, ES_adj_ = 0.00 (−0.118 –[−0.118])

^a^At follow-up (baseline adjusted).

^b^*p*-Values were computed using generalized linear models for continuous variables and negative binomial regression for dizziness frequency.

^c^Adjusted *p*-values were computed using the Holm-Bonferroni procedure.

^d^Effect sizes were computed using Cohen’s d formula; adjusted effect size = follow-up effect size minus baseline effect size.

Abbreviations: CSRT, Choice-Stepping Reaction Time; DHI, Dizziness Handicap Inventory; ES_adj_, adjusted effect size; IQR, interquartile range; N/A, not applicable; *p*_adj_, adjusted *p*; RR, relative risk.

### Effect of the multifaceted tailored intervention on secondary outcomes

The intention-to-treat analysis revealed no significant improvements in composite physiological fall risk and dynamic balance measures, anxiety, depression, neuroticism, fear of falling, or orthostatic hypotension (Table 3).

**Table 3:** Secondary outcome measures of participants in intervention and control groups at baseline and follow-up assessments.

	Baseline		Follow-up		Mean (95% CI) difference between groups^a^ or OR (95% CI), *p*^b^, ES_adj_^c^
	Control (*n* = 148)	Intervention (*n* = 146)	Control (*n* = 137)	Intervention (*n* = 137)	
**PPA, mean (SD), score**	0.78 (0.79)	0.73 (0.85)	1.06 (0.81)	0.89 (0.68)	−0.13 (−0.27 to 0.02), *p* = 0.085, ES_adj_ = 0.17 (0.23 − 0.06)
**Coordinated stability, median (IQR), mean (SD), score**	2.0 (0.0 to 8.0); 5.2 (7.8)	2.0 (0.0 to 9.0); 5.7 (8.2)	1.0 (0.0 to 5.0); 4.6 (8.1)	1.0 (0.0 to 8.0); 5.6 (9.3)	0.6 (−0.6 to 1.8), *p* = 0.327, ES_adj_ = 0.18 (0.12 − [−0.06])
**GAD-7, median (IQR), mean (SD), score**	2.0 (0.0 to 4.0); 2.4 (2.7)	2.0 (0.0 to 5.0); 3.4 (4.0)	1.0 (0.0 to 4.0); 2.5 (3.4)	2.0 (0.0 to 4.0); 2.7 (3.2)	−0.4 (−1.0 to 0.3), *p* = 0.261, ES_adj_ = 0.35 (0.06 − [−0.29])
**PHQ-9, median (IQR), mean (SD), score**	2.0 (1.0 to 5.0); 3.8 (4.4)	3.0 (1.0 to 6.0); 4.0 (4.1)	3.0 (1.0 to 5.0); 3.6 (4.2)	2.0 (1.0 to 4.0); 3.5 (4.1)	−0.3 (−0.9 to 0.3), *p* = 0.397, ES_adj_ = 0.07 (0.02 − [−0.05])
**Neuroticism, mean (SD), score**	17.2 (8.1)	19.0 (7.7)	16.6 (7.2)	17.4 (7.7)	−0.6 (−1.6 to 0.3), *p* = 0.203, ES_adj_ = 0.34 (0.11 − [−0.23])
**Icon-FES, median (IQR), mean (SD), score**	17.0 (14.0 to 22.0); 18.3 (6.9)	18.0 (14.0 to 23.0); 19.6 (7.4)	16.0 (13.0 to 20.0); 17.6 (5.7)	17.0 (13.0 to 20.0); 17.8 (6.2)	−0.4 (−1.6 to 0.7), *p* = 0.453, ES_adj_ = 0.21 (0.03 − [−0.18])
**Orthostatic hypotension, No (%)**	30 (20)	35 (24)	30 (23)	34 (26)	OR = 1.002 (0.537 to 1.869), *p* = 0.995

^a^At follow-up (baseline adjusted).

^b^*p*-Values were computed using generalized linear models for continuous variables and binary logistic regression for orthostatic hypotension at follow-up, controlling for baseline orthostatic hypotension.

^c^Effect sizes were computed using Cohen’s d formula; adjusted effect size = follow-up effect size minus baseline effect size.

Abbreviations: DHI, Dizziness Handicap Inventory; ES_adj_, adjusted effect size; GAD-7, Generalized Anxiety Disorder seven-item scale; Icon-FES, Iconographical Falls Efficacy Scale; IQR, interquartile range; OR, odds ratio; PHQ-9, Patient Health Questionnaire nine-item scale; PPA, Physiological Profile Assessment.

### Intervention-specific effects on primary and secondary outcomes

There were indications for intervention-specific improvements at the 6-month reassessment (S2–S5 Tables). Compared with the control group and controlling for baseline scores, DHI scores were significantly lower in the intervention versus the control group following vestibular rehabilitation (mean [95% CI] difference between groups [baseline adjusted]: −6.3 [−10.2 to −2.3]; *p* = 0.002), CBT (mean [95% CI] difference between groups [baseline adjusted]: −9.2 [−16.7 to −1.8]; *p* = 0.015), and medical management (mean [95% CI] difference between groups [baseline adjusted]: −4.6 [−8.4 to −0.8], *p* = 0.019). At completion of the trial and controlling for baseline performance, compared with their control counterparts, participants in the vestibular rehabilitation group also significantly improved their CSRT performance (mean [95% CI] difference between groups [baseline adjusted]: −43 [−84 to −2]; *p* = 0.040), and those in the exercise intervention group significantly reduced their composite physiological fall risk (mean [95% CI] difference between groups [baseline adjusted]: −0.37 [−0.68 to −0.06]; *p* = 0.018). At the 6-month follow-up, the CBT recipients significantly improved GAD-7 scores of anxiety (mean [95% CI] difference between groups [baseline adjusted]: −3.6 [−6.6 to −0.7]; *p* = 0.015).

## Discussion

This study investigated the effects of a multifactorial tailored intervention on dizziness handicap, frequency of dizziness episodes, and physical function in a sample of middle-aged and older people suffering from dizziness. Overall, our intervention significantly improved dizziness handicap. Given that around 12% of the community aged over 50 years report dizziness [5], using comprehensive objective assessment and individualized evidence-based interventions has the potential to offer a more effective and efficient approach to this common problem.

The need for multifactorial assessment and intervention has been apparent for some time. Nearly 2 decades ago, Sloane and Dallara advocated for the development and implementation of strategies to more effectively reduce symptoms and dizziness disability. They recommended focusing on improving functional ability to increase the quality of life when the cause of dizziness is uncertain and/or when subsequent treatment does not decrease dizziness symptoms [56]. More recently, Maarsingh and colleagues recommended a simultaneous diagnosis- and prognosis-oriented approach for dizzy older people in primary care, whereby therapies can be prescribed based on deficits identified at assessment [57,58]. However, the majority of dizziness interventions have focused on a single cause of dizziness–most often vestibular impairment [22–24,27–29,31,59,60]—with very few studies combining 2 therapies [29,30,32] despite accumulating evidence to show that dizziness becomes increasingly multifactorial in older age [18–21]. Our comprehensive baseline assessment identified 2 or more potential causes of dizziness in 39% of participants, clearly supporting the need for tailored interventions comprising evidence-based therapies as a crucial aspect of an effective dizziness management plan.

The adjusted effect size of the intervention on dizziness handicap was small (0.25) and lower than anticipated in the power calculation (0.37), and potential reasons for this are discussed in the limitations. However, given our sample’s low mean DHI score at baseline (mean [SD]: 25.4 [18.3]), a between-group mean difference of 3.7 points in DHI score is between the 10% score change suggested by Treavalen [61] as clinically relevant for this scale and the 11-point minimally important change computed by Tamber and colleagues [62]. This score difference is also similar to that reported in recent large randomized controlled trials of vestibular rehabilitation (mean between-groups difference in DHI range: 4.3 to 6.15 points) (sample sizes, *n* range: 170–337) [22,23,28]. The intervention had no significant effects on the remaining primary outcomes: frequency of dizziness episodes, CSRT, and gait variability. In retrospect, these measures may have been suboptimal choices for the interventions tested. The dizziness frequency measure proved to be fluctuating and highly variable, with participants suffering dizziness episodes of markedly different severity and ranging from no episodes over the follow-up period to omnipresent. It was therefore (a) unlikely to have been a uniform measure and (b) difficult to effect change.

The intervention-specific analyses revealed that the individual interventions were effective in managing specific aspects of dizziness, as hypothesized: fall risk (composite physiological function) for the Otago exercise programme [13,63], psychogenic dizziness-related anxiety for the CBT programme [15], and balance (CSRT) for vestibular rehabilitation [23,60]. The faster CSRTs in the group receiving vestibular rehabilitation is particularly encouraging because CSRT is a composite measure of balance, strength, and reaction time and a proxy-measure of fall risk. The improved performance in this dynamic balance test may have resulted from the incorporation of visual motion and head/eye coordination exercises along with exercises to enhance balance confidence.

The fortnightly multidisciplinary meeting was crucial to the success of the intervention because it allowed us to examine the participants’ results from an interdisciplinary perspective, which has been a major limitation of previous research in the field. We recommend that smaller clinics adopt a multidisciplinary approach to the assessment of their patients even if they do not have the specialist on site. Such approach can be done by selecting key measures from each domain.

### Strengths and limitations of the study

Strengths of the study included the broad sample of middle-aged and older people reporting dizziness, the multidisciplinary input and objective criteria for assigning participants to interventions, [33] the prospective ascertainment of dizziness episodes, the timely implementation of the interventions, and the pragmatic study design that utilised available healthcare services. The approach therefore represents a model for multidisciplinary assessment and intervention that could be incorporated into clinical practice.

We also acknowledge certain limitations. First, the single-centre nature of the study together with the reliance on self-selected volunteers as participants may limit the generalizability of the study. Second, our intervention comprised one or more individualized evidence-based components. As such, we cannot determine the effect of specific interventions, and it may be that certain interventions were key and others less efficacious. Third, in 23% of the sample, no cause of dizziness could be identified from the assessments, questionnaires, and interviews. Maarsingh and colleagues reported unclear causes of dizziness in only 8% of 417 older people comprehensively assessed in a primary care setting; however, their participants suffered from persistent dizziness [19] and accordingly reported a higher mean (SD) DHI of 36.3 (19.9). In contrast, eligibility for our study only required having suffered from at least one episode of dizziness in the past year. In addition, people receiving treatment for their dizziness at the time of baseline assessment were excluded from participating in our study; these individuals might otherwise have reported more severe dizziness and/or dizziness handicap. Low initial dizziness severity (mean [SD]: 25.4 [18.3]) leaves less room for improvement and could potentially contribute to the difficulty in establishing a diagnosis of dizziness in some of the participants with unclear causes; participants with no obvious cause of dizziness on baseline assessments (*n* = 71) had significantly lower mean DHI scores (mean [SD]: 15.8 [10.1]) than the rest of the sample (mean [SD]: 28.2 [19.1]; *p* < 0.001). Other possible reasons include but are not limited to the following: the need for the condition to be present at baseline testing (e.g., misplaced otoconia in the vestibular system producing BPPV), a previous dizziness episode triggered by severe circumstances (e.g., participant thrown to sea floor by a dumping wave) unlikely to reoccur or be reproducible in the laboratory, and a rare cause requiring specific testing (e.g., carotid massage).

Fourth, uptake of the interventions was only good (exercise, vestibular rehabilitation) to fair (CBT, Falls Clinic). Evident factors leading to non-adoption of the interventions included the receipt of healthcare through other care providers (specialist clinics and community health programmes), the requirement of a general practitioner referral letter for a Falls Clinic appointment, delays in organising the interventions, and dizziness symptoms being considered insufficiently disabling by participants to warrant action. Some participants referred to CBT declined this intervention, reporting that they expected a therapy for dizziness and not one for anxiety and depression. Comparative data regarding uptake and adherence to CBT within dizziness interventions are lacking. In fact, to our knowledge, only few randomized controlled trials to reduce dizziness have included CBT as an intervention. These trials included small sample sizes (*n* ≤ 41) and delivered CBT either on its own or integrated within a vestibular rehabilitation programme [26,30,32]. The face-to-face delivery method might have ensured good adherence and might explain why uptake and adherence were not reported in any of these trials. Future trials might consider the following strategies to improve CBT uptake: providing education on anxiety and depression and the good evidence base for CBT to effectively reduce depression and anxiety, additional follow-up (via telephone calls) from the research investigators and/or the general practitioner, together with exploration of potential barriers (prior beliefs and expectations) once the participant has signed up for the CBT course. However, in those who took up the interventions, adherence to the intervention was good (exercise, vestibular rehabilitation) to excellent (CBT), possibly owing to efforts to minimise potential barriers to participation in the interventions (i.e., appointments arranged with vestibular physiotherapist, provision of home-based exercise programme, and CBT offered in an internet-based or booklet form).

Finally, it is likely that participation in this study involving a comprehensive assessment may have reassured a number of participants, particularly those who were forwarded a report indicating no interventions were indicated, and subsequently reduced perceptions of dizziness and handicap. This is consistent with the finding that negative beliefs about the consequences of dizziness can be modified by vestibular rehabilitation therapy [64], for example, and the large body of research showing the powerful role of reassurance in healthcare [65].

## Conclusion

Our randomized controlled trial provides evidence that a multifactorial, tailored pragmatic approach, involving evidence-based therapies, is effective in improving dizziness handicap in a sample of community-living people aged 50 years and over self-reporting dizziness. Our findings therefore support the implementation of a multifactorial assessment combined with tailored interventions to reduce self-perceived disability associated with dizziness in middle-aged and older community-living people.

## Supporting information

S1 Text CONSORT checklist. CONSORT, Consolidated Standards of Reporting Trials.
(DOC)

S2 Text Published study protocol. (PDF)

S3 Text Example of monthly dizziness episodes diary. (DOCX)

S4 Text Statistical analysis plan. (DOCX)

S5 Text Sensitivity analysis of primary outcome measures with imputed data. (DOCX)

S1 Table Adverse events recorded during the study. (DOCX)

S2 Table Primary and relevant secondary outcome measures for the intervention and control participants eligible for vestibular rehabilitation, at baseline and follow-up assessments. (DOCX)

S3 Table Primary and relevant secondary outcome measures for the intervention and control participants eligible for the Otago home exercise programme, at baseline and follow-up assessments. (DOCX)

S4 Table Primary and relevant secondary outcome measures for the intervention and control participants eligible for the CBT, at baseline and follow-up assessments. CBT, cognitive-behavioural therapy.
(DOCX)

S5 Table Primary and relevant secondary outcome measures for the intervention and control participants eligible for the medical management (letter to general practitioner, Falls Clinic), at baseline and follow-up assessments. (DOCX)

S1 Data Study data including baseline descriptive measures and primary and secondary outcome measures. (XLSX)

## Abbreviations

BPPV: benign paroxysmal positional vertigo
CBT: cognitive-behavioural therapy
CONSORT: Consolidated Standards of Reporting Trials
CSRT: Choice-Stepping Reaction Time
DHI: Dizziness Handicap Inventory
GAD-7: Generalized Anxiety Disorder seven-item scale
GPCOG: General Practitioner assessment of Cognition
PHQ-9: Patient Health Questionnaire nine-item scale
RR: relative risk
