# Supplementary material for: Reducing the burden of dizziness in middle-aged and older people: A multifactorial, tailored, single-blind randomized controlled trial
Source: PLoS Med. 2018 Jul 24;15(7):e1002620. doi: 10.1371/journal.pmed.1002620 (PMC6057644; doi:10.1371/journal.pmed.1002620)
Supplement: S2 Text — (PDF) [file pmed.1002620.s002.pdf]

Menant et al. BMC Geriatrics (2017) 17:56
DOI 10.1186/s12877-017-0450-3

BMC Geriatrics

STUDY PROTOCOL Open Access

Tailored multifactorial intervention to improve dizziness symptoms and quality of life, balance and gait in dizziness sufferers aged over 50 years: protocol for a randomised controlled trial

Jasmine C. Menant[1,2], Americo A. Migliaccio[1], Cameron Hicks[1], Joanne Lo[1], Daniela Meinrath[1,2], Mayna Ratanapongleka[1], Jessica Turner[1], Daina L. Sturnieks[1], Kim Delbaere[1,2], Nickolai Titov[3], Catherine McVeigh[4], Jacqueline C. T. Close[1,4] and Stephen R. Lord[1,2*]

Abstract

Background: Dizziness is a frequently reported symptom in older people that can markedly impair quality of life. This manuscript presents the protocol for a randomised controlled trial, which has the main objective of determining the impact of comprehensive assessment followed by a tailored multifaceted intervention in reducing dizziness episodes and symptoms, improving associated impairments to balance and gait and enhancing quality of life in older people with self-reported significant dizziness.

Methods: Three hundred people aged 50 years or older, reporting significant dizziness in the past year will be recruited to participate in the trial. Participants allocated to the intervention group will receive a tailored, multifaceted intervention aimed at treating their dizziness symptoms over a 6 month trial period. Control participants will receive usual care. The primary outcome measures will be the frequency and duration of dizziness episodes, dizziness symptoms assessed with the Dizziness Handicap Inventory, choice-stepping reaction time and step time variability. Secondary outcomes will include health-related quality of life measures, depression and anxiety symptoms, concern about falling, balance and risk of falls assessed with the physiological fall risk assessment. Analyses will be by intention-to-treat.

Discussion: The study will determine the effectiveness of comprehensive assessment, combined with a tailored, multifaceted intervention on dizziness episodes and symptoms, balance and gait control and quality of life in older people experiencing dizziness. Clinical implications will be evident for the older population for the diagnosis and treatment of dizziness.

Trial registration: The study is registered with the Australia New Zealand Clinical Trials Registry ACTRN12612000379819.

Keywords: Dizziness, Vertigo, Vestibular, Light-headedness, Randomised controlled trial, Aged, Accidental falls, Postural balance

* Correspondence: s.lord@neura.edu.au
[1]Neuroscience Research Australia, University of New South Wales, Sydney, Australia
[2]School of Public Health & Community Medicine, University of New South Wales, Sydney, Australia
Full list of author information is available at the end of the article

© The Author(s). 2017 **Open Access** This article is distributed under the terms of the Creative Commons Attribution 4.0 International License (http://creativecommons.org/licenses/by/4.0/), which permits unrestricted use, distribution, and reproduction in any medium, provided you give appropriate credit to the original author(s) and the source, provide a link to the Creative Commons license, and indicate if changes were made. The Creative Commons Public Domain Dedication waiver (http://creativecommons.org/publicdomain/zero/1.0/) applies to the data made available in this article, unless otherwise stated.

Background

Dizziness is a frequent complaint of older people. Current or chronic symptoms of dizziness are reported by 10% [1, 2] to 30% [3, 4] of community-dwelling older adults with an increasing prevalence as people age [1, 3, 5]. Dizziness markedly impairs quality of life [6], and is associated with a two-fold increase in the prevalence of self-reported functional disability [1], worsening of depressive symptoms [2, 4], decreased participation in social activities, poor self-reported health and reduced falls self-efficacy [4]. There is also evidence of a relationship between acute dizziness and falls [3, 7] and the frequency of dizziness episodes is associated with disability [1], falls and syncopal events [7].

Dizziness is a subjective sensation that is used to describe feelings such as light-headedness, feeling faint, head spinning, room spinning, unsteadiness, and feeling woozy or giddy. Clinically, dizziness is described as a feeling of altered orientation in space [8]. Traditionally, it has been divided into four main subtypes [9, 10]: (i) vertigo, where patients express illusory sensation of self-motion which often is the result of a vestibular system disorder; (ii) presyncopal dizziness, a light-headed sensation associated with cerebral hypoperfusion; (iii) psychogenic dizziness, associated with a mental health issue such as generalised anxiety; and (iv) disequilibrium and non-specific dizziness, often associated with neuromuscular causes. In older people, dizziness may have a multifactorial aetiology, with many dizzy older patients fulfilling criteria for two or more of the subtypes described above [11–13].

This multifactorial aetiology [11–14] combined with imprecise symptom descriptions [15], make it difficult to establish an accurate diagnosis of dizziness [16, 17] A retrospective chart audit of 50 older patients with dizziness from a family practice showed that 45% did not have a diagnosis and 10% had more than one diagnosis [16]. Furthermore, in 20% of consecutive older patients presenting to a general practice, there was no attributable cause of dizziness based on clinical characteristics [17]. Other contributing factors to the diagnostic challenge of dizziness include overreliance on symptom description to establish a diagnosis [18], a tendency to diagnose conditions in the clinicians own field of experience (“the blind men and the elephant” phenomenon) [19], and the lack of diagnostic accuracy of many tests [20].

If an accurate diagnosis for the underlying cause of dizziness can be made, there is potential for effective therapy. The Epley manoeuvre and vestibular rehabilitation appear effective in managing Benign Paroxysmal Positional Vertigo (BPPV) [21] and some unilateral peripheral disorders [22], respectively. There are also well-established syncope assessment and management guidelines [23], fall prevention balance and strength exercise programs for older people [24, 25], and emerging cognitive behavioural therapies (CBT) that could be readily applied to older patients with psychogenic dizziness [26].

A comprehensive multidisciplinary assessment of dizziness holds great promise for more effectively diagnosing and treating dizziness in older people. There have been models proposed before and these have had some success in reducing the number of unresolved cases [5, 9, 13, 14, 27, 28]. However, these assessments are either very time consuming [9], have never been further developed as tools [13, 27], or are not based on empirical evidence [14, 27, 28]. A one-stop dizziness assessment would be welcomed by older people with dizziness who report that they would like to minimize the number of specialist referrals, for logistical reasons, cost and because they perceive that specialist’s consultations often do not result in successful treatment [29].

This randomised controlled trial will aim to improve the understanding of dizziness, its assessment and its management in older people, overcoming the barriers we have described. We have designed a comprehensive, multidisciplinary battery of vestibular, cardiovascular, neuromuscular, balance and psychological assessments to improve the likelihood of obtaining a diagnosis for the symptom of dizziness in middle-aged and older people.

Primary objective

To compare the effect of a tailored multifaceted intervention with usual care on frequency of dizziness episodes, balance and gait performance and quality of life in people aged 50 years and over with self-reported significant dizziness.

Secondary objectives

- To establish the cost-effectiveness of the program from the health provider’s perspective.
- To determining the effects of the program on vestibular, cardiovascular and psychological measures associated with dizziness.

Methods

Design

A single blind parallel group randomised controlled trial will be conducted in 300 participants with dizziness. Figure 1 presents the study flow diagram. The Human Research Ethics Committee of the University of New South Wales approved this study (HC 12152). The study is registered with the Australia New Zealand Clinical Trials Registry (ACTRN12612000379819) (Table 1).

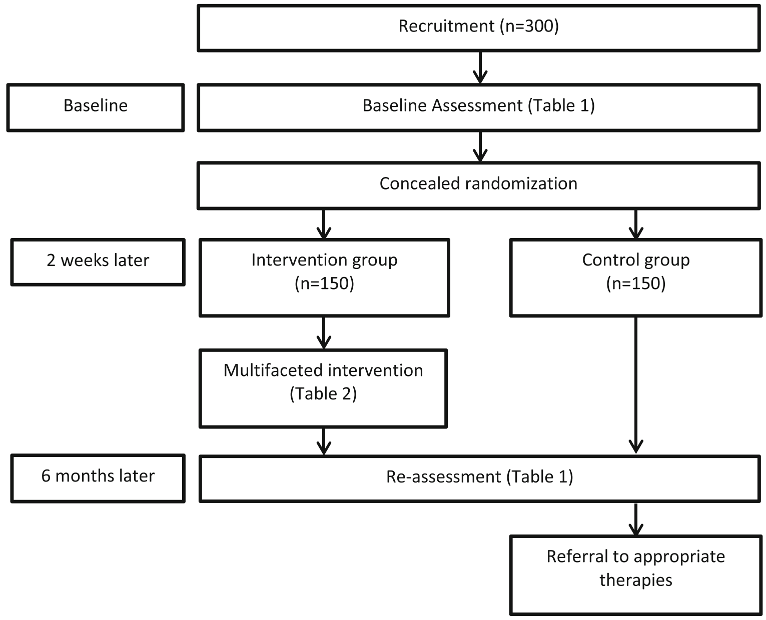

Fig. 1 Study flow diagram

Participants

People aged 50 years or older, living in the community will be recruited through advertisements; flyers on community facilities, hospital and university noticeboards; articles in newspapers and newsletters for older people; the Neuroscience Research Australia (NeuRA) website, newsletter and mailing list; and by mail box drops within the local community and retirement villages. To be eligible for the study, participants must: (i) be aged 50 years and over; (ii) have experienced at least one significant episode of dizziness in the past 12 months; (iii) live independently in the community or retirement village and; (iv) be able to understand English. Data will be collected in Australia.

People will be ineligible to participate in the study if they; (i) have a degenerative neurological condition; (ii) are currently receiving treatment for their dizziness; (iii) have a cognitive impairment (a General Practitioner Assessment of Cognition (GPCOG) of <5) [30] and/or; (iv) are unable to walk 20 m without difficulty with the use of a walking aid.

Participants identified on assessment with conditions that require urgent treatment defined as suspected stroke, transient ischemic attack or other undiagnosed neurological or acute cardiovascular condition, severe depressive or anxiety symptoms will also be excluded from the study and referred following consent for appropriate treatment.

Baseline assessment and case conference

All eligible participants will attend NeuRA for a three-hour baseline assessment undertaken by trained research assistants. The study will be explained in detail and written consent obtained prior to commencing the assessment. The assessments are outlined in Table 2. These include diagnostic tests for descriptive purposes and for allocating intervention participants to treatment arms as well as baseline measures for the primary and secondary outcomes.

A multidisciplinary case conference will then be held within two weeks of the assessment to reach a consensus diagnosis and design a tailored intervention plan based on the baseline assessment results. Results of all assessments undertaken will be reported and recommendations regarding appropriate therapies derived and prioritised. Panel members will include at least a geriatrician, a vestibular neuroscientist, an exercise physiologist and a baseline assessor.

Randomisation

After completion of the baseline assessment and case conference, participants will be randomised into intervention or control groups. Permuted blocks (sizes: 2, 4 and 6) using a computer generated random number schedule will be performed to determine randomisation order. Allocation will be concealed by using central randomisation performed by NeuRA personnel not otherwise involved in the study. Staff performing outcome measurement and data analysis for the primary outcomes will be blinded to group allocation. However, due to the nature of the intervention, it is not possible to blind the staff administering interventions or the participants. Participants will be instructed not to inform the assessors of their intervention status.

Table 1 Trial registration data from the World Health Organization Trial Registration Data Set

Data category	Information
Primary registry and trial identifying number	anzctr.org.au<br>ACTRN12612000379819
Date of registration in primary registry	3 April 2012
Secondary identifying numbers	N/A
Source(s) of monetary or material support	Australian National Health and Medical Research Council
Primary sponsor	Professor Stephen Lord, Neuroscience Research Australia
Secondary sponsor	Dr Jasmine Menant, Neuroscience Research Australia
Contact for public queries	Professor Stephen Lord, DSc [S.Lord@neura.edu.au]
Contact for scientific queries	Professor Stephen Lord, DSc<br>Neuroscience Research Australia, Randwick Sydney, Australia
Public title	Treating dizziness in older people
Scientific title	A randomised controlled trial of dizziness interventions based on a multidisciplinary assessment in older people: towards the development of a multiple profile assessment of dizziness – the MPA-D
Countries of recruitment	Australia
Health condition(s) or problem(s) studied	Dizziness in older people
Intervention(s)	Active comparator: Multifaceted tailored intervention including a home exercise program, a vestibular rehabilitation program, a booklet-based cognitive behavioural therapy, and/or a comprehensive geriatric assessment and medication review.
	Placebo comparator: usual care, no intervention
Key inclusion and exclusion criteria	Ages eligible for study: ≥50 years<br>Sexes eligible for study: both<br>Accepts healthy volunteers: yes
	Inclusion criteria: adult patient (≥50 years), having experienced one or more episode(s) of dizziness in the past year and not being currently treated for it; living independently in the community or retirement village; able to understand English.
	Exclusion criteria: presence of a diagnosed degenerative neurological condition or severe cognitive impairment (GP-Cog score equal or below 4).
Study type	Interventional
	Allocation: randomized intervention model. Parallel assignment masking: single blind (investigator, outcomes assessor)
	Primary purpose: prevention
	Phase III
Date of first enrolment	August 2012
Target sample size	300
Recruitment status	Completed
Primary outcome(s)	• Frequency and duration of dizziness episodes experienced in the 6-months period between baseline and re-test.<br>• Dizziness-related quality of life recorded using a validated questionnaire, the Dizziness Handicap Inventory<br>• Choice stepping reaction time: a composite measure of reaction time, strength and balance.<br>Time points for all outcomes: baseline and 6 months post-baseline (i-e at the end of the intervention period)
Key secondary outcomes	• Tilt table test of orthostatic hypotension<br>• Coordinated Stability - a test of leaning balance<br>• Geriatric depression scale - 15 items: a questionnaire to assess depression disorders<br>Time points for all outcomes: baseline and 6 months post-baseline (i-e at the end of the intervention period)

Table 2 Baseline assessment (BA) and re-assessment (RA) including outcome measures

Domain	Test and criterion for abnormal/impaired performance	BA	RA	O
General	• Patients' history and medications.	✓	✓	-
	• Frequency and duration of dizziness episodes.	✓	✓[a]	P
	• Dizziness Handicap Inventory [36].	✓	✓	P
	• Vertigo Symptom Scale [37].	✓	✓	S
	• EQ-5D: a generic measure of health status which will be used in the economic evaluation [38].	✓	✓	S
	• AQOL-6D (Assessment of Quality of Life): a utility measure of quality of life - will be used to conduct the economic evaluation [39].	✓	✓	S
Vestibular	• Eye movement examination (nystagmus, pursuit, saccades, Vestibulo-Ocular Reflex (VOR) suppression) using Frenzel's glasses [40] – abnormalities are indicative of peripheral and/or central vestibular disorder.	✓	✓	-
	• Dix-Hallpike positional manoeuvre : a positive test indicates Benign Paroxysmal Positional Vertigo [22].	✓	✓	-
	• Dynamic-Visual Acuity (DVA) Test – assessed during passive head impulses, score > 0.316 indicates impaired DVA [41]	✓	✓	-
	• Video Head Impulse Test: a + test indicates impaired VOR, peripheral vestibular disorders [42–44].	✓	✓	-
	• Rotary chair testing: VOR gain < 0.75 (at 1 Hz) and time constant < 6 s indicates VOR hypofunction, time constant >12 s indicates cerebellum disorders [41]	✓	✓	-
Cardio-vascular	• Tilt Table test - a decrease in systolic blood pressure ≥20 mmHg or a decrease of systolic blood pressure to ≤90 mmHg after 3 min of upright standing, defines orthostatic hypotension whether or not symptoms occur [23, 45, 46].	✓	✓	S
	• Electrocardiogram - arrhythmia defines possible arrhythmia-related syncope [23].	✓	-	-
Balance, Gait and Fall Risk	• Physiological Profile Assessment of fall risk - performances 1 standard deviation below established norms for older people aged 65 years and over define impairments in vision, touch, peripheral sensation, reaction time, lower limb strength and balance [47, 48].	✓	✓	S
	• Coordinated Stability - score ≥15 error points indicates impaired dynamic balance [7, 49].	✓	✓	S
	• Choice-Stepping Reaction Time - total reaction time > 1.4 s indicates impaired performance (composite measure of strength, balance and reaction time) [31].	✓	✓	P
	• Step timing variability at preferred walking speed > 0.03 s indicates impaired walking stability [32, 50].	✓	✓	P
Psychological	• Falls Efficacy Scale –International - score > 22 indicates high levels of fear of falling [51, 52].	✓	✓	S
	• Patient Health Questionnaire –9 - score > 9 indicates depressive symptoms [53–56].	✓	✓	S
	• Generalized Anxiety Disorder 7 items scale - score > 7 indicates anxiety disorders [57].	✓	✓	S
	• Neuroticism scale of the NEO-Five Factor Inventory– score ≥ 56 indicates a high level of neuroticism [58].	✓	✓	S

BA Baseline Assessment, *RA* Re-assessment, *O* Outcome measure, *S* secondary, *P* Primary
[a]assessed monthly throughout the six-month trial period

The intervention

Based on their underlying conditions and case conference recommendations, appropriate interventions will be organised for participants assigned to the intervention group (Table 3). The intervention plan will be guided by published normative data for the vision, sensorimotor, balance and psychological tests and the presence of abnormal results in our vestibular and cardiovascular tests. Due to the multifactorial aetiology of dizziness in older people, it is likely that many participants will require multiple interventions which we will implement in a staged manner.

Adverse events (for example, a fall during an exercise session) will be monitored with monthly calendars and telephone calls as required and adherence to all interventions documented with therapist records and/or participant diaries.

A trial safety committee composed of two Senior staff of Neuroscience Research Australia (a Senior scientist and a Senior biostatistician), not involved in any aspect of the study and independent from the sponsor and investigators, will review the withdrawal, deceased and incident data of the trial annually and report to the Ethics Committee.

The control group

Control group participants will receive usual care during the six-month trial period. At completion of the trial, they will be provided with their baseline and reassessment reports and given the option to being referred appropriate therapies.

Primary and secondary outcomes

The primary outcome measures outlined in Table 2 will capture the four crucial aspects of the trial: dizziness, balance, walking stability and quality of life. The secondary outcome measures, also outlined in Table 2, will elucidate

Table 3 Underlying conditions diagnosed and indicated interventions

Underlying condition	Intervention
Benign Paroxysmal Positional Vertigo	Epley's manoeuvre [22]: repositioning manoeuvre performed on the patient by a physiotherapist to remove vestibular debris from the semi-circular canals.
Peripheral vestibular conditions	1. Vestibular rehabilitation [22] administered by a vestibular physiotherapist.<br>2. Referral to an ear-nose-throat specialist or neurologist as indicated.
Central vestibular conditions	1. Onward referral to neurologist, magnetic resonance imaging scan/other scans as indicated.<br>2. Review at Falls Clinic by Consultant Geriatrician for management of any co-morbidities and medication review.
Anxiety/depression/low falls efficacy	1. Online or booklet-based Cognitive Behavioural Therapy (CBT) with telephone support to a clinical psychologist; the program will be focusing on management of anxiety, depression and fear of falling. The booklet will have the same contents and presentation as the internet-based CBT [59–61]; given that not all participants might have access to internet in their home, the booklet-based CBT will ensure that all participants from the intervention group who require a therapy addressing psychogenic dizziness will receive the same CBT intervention. The CBT program will involve 5 lessons including a weekly homework assignment over 8 weeks.<br>2. Referral to other mental health services if indicated.
Cardiovascular conditions	1. Medication management of postural hypotension [23]: the participant will be referred to a Consultant Geriatrician's Falls Clinic for a medication review.<br>2. Onward referral to cardiologist if indicated.
Balance, strength, and gait impairments	Otago Exercise Program [24, 62, 63]: this home-based exercise program consists of resistance training and balance training exercises; it will involve individualised prescription of exercise in 5 home visits by a physiotherapist and monthly follow-up phone calls. Participants will be encouraged to exercise 3 times a week (approximately 30 min each time) for 6 months.
Vision/sensation impairments	1. Review at Falls Clinic by a Consultant Geriatrician.<br>2. Onward referral to neurologist/ophthalmologist/optometrist if indicated.

how the interventions assist in ameliorating dizziness symptoms. Dizziness episodes and duration will be ascertained with monthly calendars and telephone calls as required. Participants will return to NeuRA for reassessment of outcomes at the end of the trial (6 months post randomisation). Staff monitoring the dizziness episodes and administering reassessments will be blinded to group allocation.

Sample size calculation

A power analysis determined that 300 participants (150 per group) will need to be recruited to provide 80% power to detect a statistically significant 20% between-group difference in the primary outcome measures. For these calculations, we assumed an alpha of 0.05 and a dropout rate of 15%. We assumed the following control group means (standard deviations), based on values from previous studies of older people [31–33]: choice-stepping reaction time = 1322 (331) ms, step-time variability = 0.02 (0.01) s, and dizziness handicap inventory = 37 (2).

Statistical analysis

All analyses will use an intention-to-treat approach and analysis of the primary outcomes will be conducted masked to group allocation. Dizziness episodes will be contrasted between groups with negative binomial regression due to the likely Poisson- like distribution of this variable. Between-group comparisons of retest performance for the continuously-scored primary and secondary outcome measures will be made using General Linear Models (ANCOVA) controlling for pre-test performance and other potential confounders. Estimates of the magnitude of clinical improvement in symptoms of anxiety and depression will be derived using calculations of effect size (Cohen's d) and percentage of symptom improvement. Predictors of uptake, acceptability and adherence will be established using multivariate modelling techniques including multiple linear and logistic regression analyses. Analyses will be conducted using the SPSS software package.

Economic analysis

An economic evaluation will be conducted from the perspective of the health and community service provider. Benefits will be measured in terms of quality-adjusted life years (QALY) gained, based on utility weights derived from the EQ-5D and AQoL-6D collected at baseline and at 6 months. The study will collect data on the cost to deliver the intervention program. Using the mean costs in each trial arm, and the mean health outcomes in each arm, the incremental cost per QALY of the intervention group compared to control groups will be calculated and results plotted on a cost-effectiveness plane.

Bootstrap sampling will be used to estimate a distribution around costs and health outcomes, and to calculate the confidence intervals around the incremental cost-effectiveness ratio. We will conduct a one-way sensitivity analysis and a probabilistic sensitivity analysis to estimate the joint uncertainty in cost and effect parameters.

A cost-effectiveness acceptability curve will be plotted. A cost-effectiveness acceptability curve provides information about the probability that an intervention is cost-effective, given a decision makers' willingness to pay for each additional QALY.

Discussion

This trial is expected to improve our understanding of dizziness by identifying the main contributory causes of this prevalent and debilitating condition in a representative sample of older people. We also hope to demonstrate the efficacy of conducting a comprehensive assessment followed by a tailored multifaceted intervention in reducing dizziness symptoms, improving associated impairments to balance and gait and enhancing quality of life in older people with self-reported dizziness symptoms. If successful, the findings of this trial could be readily translated into clinical practice. The trial also has the capacity to drive the implementation of a profile-based assessment and intervention based on empirical data from quantitative tests that best identify dizziness subtypes.

The outcomes of this study may have important implications, not only for the older population, but also for the general population suffering with dizziness, as it is documented that two percent of adults seek medical attention annually for a new symptom of moderate to severe dizziness or vertigo [34]. Unintended consequences resulting from a sub-optimal assessment of dizziness include multiple referrals to specialists, abandonment of social activities, loss of independence, increased sick leave and job loss, depression and anxiety, falls and concern about falls [1–4, 34, 35]. A validated and multifaceted dizziness profile assessment complemented by evidence-based therapies has the potential to improve individual quality of life across all ages and clinician quality of care, as well as reduce health care and community costs.

Conclusions

This study will determine the impact of a comprehensive assessment followed by a tailored multifaceted intervention in reducing dizziness episodes and symptoms, improving associated impairments to balance and gait and enhancing quality of life in older people with self-reported significant dizziness.

Acknowledgement
Not applicable.

Funding
This trial is funded by an Australian National Health and Medical Research Council (Reference number 1026726). The salaries of KD and SL are funded by Australian National Health and Medical Research Council fellowships. The salary of AAM is funded by a Garnett Passe & Rodney Williams Memorial Foundation Senior Principal Research Fellowship. The research is conducted independently from the funding body.

Availability of data and materials
Not applicable.

Authors' contributions
JM and SL drafted the manuscript. All authors are actively involved in the study. All authors read and approved the final manuscript. SL is the primary study sponsor.

Competing interests
The authors declare that they have no competing interests.

Consent for publication
Not applicable.

Ethics approval and consent to participate
The Human Research Ethics Committee of the University of New South Wales approved this study (HC 12152). Written consent was obtained from all participants prior to commencing the study.

Author details
[1]Neuroscience Research Australia, University of New South Wales, Sydney, Australia. [2]School of Public Health & Community Medicine, University of New South Wales, Sydney, Australia. [3]Department of Psychology, Macquarie University, Sydney, Australia. [4]Prince of Wales Clinical School, Sydney, NSW, Australia.

Received: 5 November 2016 Accepted: 11 February 2017
Published online: 15 February 2017

References

1. Aggarwal NT, Bennett DA, Bienias JL, de Leon Mendes CF, Morris MC, Evans DA. The prevalence of dizziness and its association with functional disability in a biracial community population. J Gerontol A Biol Sci Med Sci. 2000;55:M288–92.
2. Stevens KN, Lang IA, Guralnik JM, Melzer D. Epidemiology of balance and dizziness in a national population: findings from the English Longitudinal Study of Ageing. Age Ageing. 2008;37:300–5.
3. Colledge NR, Wilson JA, Macintyre CC, MacLennan WJ. The prevalence and characteristics of dizziness in an elderly community. Age Ageing. 1994;23:117–20.
4. Tinetti ME, Williams CS, Gill TM. Health, functional, and psychological outcomes among older persons with chronic dizziness. J Am Geriatr Soc. 2000;48:417–21.
5. Maarsingh OR, Dros J, Schellevis FG, van Weert HC, Bindels PJ, Horst HE. Dizziness reported by elderly patients in family practice: prevalence, incidence, and clinical characteristics. BMC Fam Pract. 2010;11:2.
6. Neuhauser HK, von Brevern M, Radtke A, Lezius F, Feldmann M, Ziese T, et al. Epidemiology of vestibular vertigo: a neurotologic survey of the general population. Neurology. 2005;65:898–904.
7. Delbaere K, Close JCT, Heim J, Sachdev PS, Brodaty H, Slavin MJ, et al. A multifactorial approach for understanding fall risk in older people - a decision tree model. J Am Geriatr Soc. 2010;58:1679–85.
8. Halmagyi GM, Baloh R. Overview of common syndromes of vestibular disease. In: Baloh R, Halmagyi GM, editors. Disorders of the vestibular system. New York: Oxford University Press; 1996. p. 291–9.
9. Drachman DA, Hart CW. An approach to the dizzy patient. Neurology. 1972;22:323–34.
10. Baloh RW. Dizziness in older people. J Am Geriatr Soc. 1992;40:713–21.
11. Sloane PD, Coeytaux RR, Beck RS, Dallara J. Dizziness: state of the science. Ann Intern Med. 2001;134:823–32.
12. Tinetti ME, Williams CS, Gill TM. Dizziness among older adults: a possible geriatric syndrome. Ann Intern Med. 2000;132:337–44.
13. Kroenke K, Lucas CA, Rosenberg ML, Scherokman B, Herbers Jr JE, Wehrle PA, et al. Causes of persistent dizziness. A prospective study of 100 patients in ambulatory care. Ann Intern Med. 1992;117:898–904.
14. Maarsingh OR, Dros J, Schellevis FG, van Weert HC, van der Windt DA, ter Riet G, et al. Causes of persistent dizziness in elderly patients in primary care. Ann Fam Med. 2010;8:196–205.
15. Newman-Toker DE, Cannon LM, Stofferahn ME, Rothman RE, Hsieh YH, Zee DS. Imprecision in patient reports of dizziness symptom quality:

a cross-sectional study conducted in an acute care setting. Mayo Clin Proc. 2007;82:1329–40.
16. Kwong EC, Pimlott NJ. Assessment of dizziness among older patients at a family practice clinic: a chart audit study. BMC Fam Pract. 2005;6:2.
17. Lawson J, Fitzgerald J, Birchall J, Aldren CP, Kenny RA. Diagnosis of geriatric patients with severe dizziness. J Am Geriatr Soc. 1999;47:12–7.
18. Stanton VA, Hsieh YH, Camargo Jr CA, Edlow JA, Lovett PB, Goldstein JN, et al. Overreliance on symptom quality in diagnosing dizziness: results of a multicenter survey of emergency physicians. Mayo Clin Proc. 2007;82:1319–28.
19. Sloane PD, Dallara J. Clinical research and geriatric dizziness: the blind men and the elephant. J Am Geriatr Soc. 1999;47:113–4.
20. Dros J, Maarsingh OR, van der Horst H, Bindels PJ, Ter Riet G, van Weert HC. Tests used to evaluate dizziness in primary care. CMAJ. 2010;182:E621–31.
21. Furman JM, Cass SP. Benign paroxysmal positional vertigo. N Engl J Med. 1999;341:1590–6.
22. McDonnell MN, Hillier SL. Vestibular rehabilitation for unilateral peripheral vestibular dysfunction. Cochrane Database Syst Rev. 2015;1:CD005397.
23. Brignole M, Alboni P, Benditt DG, Bergfeldt L, Blanc JJ, Bloch Thomsen PE, et al. Guidelines on management (diagnosis and treatment) of syncope–update 2004. Europace. 2004;6:467–537.
24. Campbell AJ, Robertson MC, Gardner MM, Norton RN, Tilyard MW, Buchner DM. Randomised controlled trial of a general practice programme of home based exercise to prevent falls in elderly women. BMJ. 1997;315:1065–9.
25. Howe TE, Rochester L, Neil F, Skelton DA, Ballinger C. Exercise for improving balance in older people. Cochrane Database Syst Rev. 2011;11:CD004963.
26. Butler AC, Chapman JE, Forman EM, Beck AT. The empirical status of cognitive-behavioral therapy: a review of meta-analyses. Clin Psychol Rev. 2006;26:17–31.
27. Colledge NR, Barr-Hamilton RM, Lewis SJ, Sellar RJ, Wilson JA. Evaluation of investigations to diagnose the cause of dizziness in elderly people: a community based controlled study. BMJ. 1996;313:788–92.
28. Maarsingh OR, Dros J, van Weert HC, Schellevis FG, Bindels PJ, van der Horst HE. Development of a diagnostic protocol for dizziness in elderly patients in general practice: a Delphi procedure. BMC Fam Pract. 2009;10:12.
29. Kruschinski C, Theile G, Dreier SD, Hummers-Pradier E. The priorities of elderly patients suffering from dizziness: a qualitative study. Eur J Gen Pract. 2010;16:6–11.
30. Brodaty H, Pond D, Kemp NM, Luscombe G, Harding L, Berman K, Huppert FA. The GPCOG: a new screening test for dementia designed for general practice. J Am Geriatr Soc. 2002;50:530–4.
31. Lord SR, Fitzpatrick RC. Choice stepping reaction time: a composite measure of falls risk in older people. J Gerontol A Biol Sci Med Sci. 2001;56:M627–32.
32. Callisaya ML, Blizzard L, Schmidt MD, McGinley JL, Srikanth VK. Ageing and gait variability–a population-based study of older people. Age Ageing. 2010;39:191–7.
33. Hansson EE, Mansson NO, Ringsberg KA, Hakansson A. Falls among dizzy patients in primary healthcare: an intervention study with control group. Int J Rehabil Res. 2008;31:51–7.
34. Neuhauser HK, Radtke A, von Brevern M, Lezius F, Feldmann M, Lempert T. Burden of dizziness and vertigo in the community. Arch Intern Med. 2008; 168:2118–24.
35. Neuhauser HK, Radtke A, von Brevern M, Feldmann M, Lezius F, Ziese T, et al. Migrainous vertigo: prevalence and impact on quality of life. Neurology. 2006;67:1028–33.
36. Jacobson GP, Newman CW. The development of the dizziness handicap inventory. Arch Otolaryngol Head Neck Surg. 1990;116:424–7.
37. Yardley L, Masson E, Verschuur C, Haacke N, Luxon L. Symptoms, anxiety and handicap in dizzy patients: development of the vertigo symptom scale. J Psychosom Res. 1992;36:731–41.
38. Rabin R, de Charro F. EQ-5D: a measure of health status from the EuroQol Group. Ann Med. 2001;33:337–43.
39. Richardson J, Day N, Peacock S, Lezzi A. Measurement of the quality of life for economic evaluation and the Assessment of Quality of Life (AQoL) Mark 2 instrument. Aust Econ Rev. 2004;37:62–88.
40. Bronstein A, Lempert T. Dizziness. A practical approach to diagnosis and management. Cambridge: Cambridge Universtiy Press; 2007.
41. Chau AT, Menant JC, Hubner PP, Lord SR, Migliaccio AA. Prevalence of vestibular disorder in older people who experience dizziness. Front Neurol. 2015;6:268.
42. Migliaccio AA, Della Santina CC, Carey JP, Niparko JK, Minor LB. The vestibulo-ocular reflex response to head impulses rarely decreases after cochlear implantation. Otol Neurotol. 2005;26:655–60.
43. Migliaccio AA, Minor LB, Carey JP. Vergence-mediated modulation of the human angular vestibulo-ocular reflex is unaffected by canal plugging. Exp Brain Res. 2008;186:581–7.
44. Schubert MC, Tusa RJ, Grine LE, Herdman SJ. Optimizing the sensitivity of the head thrust test for identifying vestibular hypofunction. Phys Ther. 2004; 84:151–8.
45. Kenny RA, Ingram A, Bayliss J, Sutton R. Head-up tilt: a useful test for investigating unexplained syncope. Lancet. 1986;1:1352–5.
46. Consensus statement on the definition of orthostatic hypotension, pure autonomic failure, and multiple system atrophy. The Consensus Committee of the American Autonomic Society and the American Academy of Neurology. Neurology. 1996;46:1470.
47. Lord SR, Menz HB, Tiedemann A. A physiological profile approach to falls risk assessment and prevention. Phys Ther. 2003;83:237–52.
48. Lord SR, Ward JA, Williams P, Anstey KJ. Physiological factors associated with falls in older community-dwelling women. J Am Geriatr Soc. 1994;42: 1110–7.
49. Lord SR, Ward JA, Williams P. Exercise effect on dynamic stability in older women: a randomized controlled trial. Arch Phys Med Rehabil. 1996;77:232–6.
50. Hausdorff JM, Rios DA, Edelberg HK. Gait variability and fall risk in community-living older adults: a 1-year prospective study. Arch Phys Med Rehabil. 2001;82:1050–6.
51. Delbaere K, Close JC, Mikolaizak AS, Sachdev PS, Brodaty H, Lord SR. The Falls Efficacy Scale International (FES-I). A comprehensive longitudinal validation study. Age Ageing. 2010;39:210–6.
52. Yardley L, Beyer N, Hauer K, Kempen G, Piot-Ziegler C, Todd C. Development and initial validation of the Falls Efficacy Scale-International (FES-I). Age Ageing. 2005;34:614–9.
53. Spitzer RL, Kroenke K, Williams JB. Validation and utility of a self-report version of PRIME-MD: the PHQ primary care study. Primary care evaluation of mental disorders. Patient health questionnaire. JAMA. 1999;282:1737–44.
54. Wiltink J, Tschan R, Michal M, Subic-Wrana C, Eckhardt-Henn A, Dieterich M, et al. Dizziness: anxiety, health care utilization and health behavior–results from a representative German community survey. J Psychosom Res. 2009; 66:417–24.
55. Kroenke K, Spitzer RL, Williams JB. The PHQ-9: validity of a brief depression severity measure. J Gen Intern Med. 2001;16:606–13.
56. Persoons P, Luyckx K, Desloovere C, Vandenberghe J, Fischler B. Anxiety and mood disorders in otorhinolaryngology outpatients presenting with dizziness: validation of the self-administered PRIME-MD Patient Health Questionnaire and epidemiology. Gen Hosp Psychiatry. 2003;25:316–23.
57. Spitzer RL, Kroenke K, Williams JB, Lowe B. A brief measure for assessing generalized anxiety disorder: the GAD-7. Arch Intern Med. 2006;166:1092–7.
58. Costa PT, McCrae RR. NEO PI-R Professional Manual. Revised NEO Personality Inventory (NEO PI-R) and NEO Five-Factor Inventory (NEO-FFI). Odessa: Psychological Assessment Resources Inc.; 1992.
59. Dear BF, Zou JB, Ali S, Lorian CN, Johnston L, Sheehan J, et al. Clinical and cost-effectiveness of therapist-guided internet-delivered cognitive behavior therapy for older adults with symptoms of anxiety: a randomized controlled trial. Behav Ther. 2015;46:206–17.
60. Staples LG, Fogliati VJ, Dear BF, Nielssen O, Titov N. Internet-delivered treatment for older adults with anxiety and depression: implementation of the Wellbeing Plus Course in routine clinical care and comparison with research trial outcomes. 2016.
61. Titov N, Fogliati VJ, Staples LG, Gandy M, Johnston L, Wootton BM, et al. Treating anxiety and depression in older adults: randomised controlled trial comparing guided v. self-guided internet-delivered cognitive-behavioural therapy. B J Psych Open. 2016;2:50–8.
62. Campbell AJ, Robertson MC, Gardner MM, Norton RN, Buchner DM. Falls prevention over 2 years: a randomized controlled trial in women 80 years and older. Age Ageing. 1999;28:513–8.
63. Gardner MM, Buchner DM, Robertson MC, Campbell AJ. Practical implementation of an exercise-based falls prevention programme. Age Ageing. 2001;30:77–83.
