# Supplementary material for: Reducing the burden of dizziness in middle-aged and older people: A multifactorial, tailored, single-blind randomized controlled trial
Source: PLoS Med. 2018 Jul 24;15(7):e1002620. doi: 10.1371/journal.pmed.1002620 (PMC6057644; doi:10.1371/journal.pmed.1002620)
Supplement: S3 Text — (DOCX) [file pmed.1002620.s003.docx]

**Dizziness Questionnaire – October 2012**

| 1. **In the past month, have you felt dizzy at all?** |
| --- |

🞎 **NO,** I have **not** experienced dizziness, vertigo or light-headedness this month 🡪Go to Question 2

🞎 **YES** 🡪 please indicate the day(s) you felt dizzy on the calendar below

**Dizziness** in the month of **October 2012**

**Please mark the calendar for each day you feel the following:
V –** Vertigo (Feeling that my head/the room was spinning around)
**L –** Lightheadedness (Feeling faint)

**D –** Other dizziness. Please specify: ____________________________________________

| **SUN** | **MON** | **TUE** | **WED** | **THUR** | **FRI** | **SAT** |
| --- | --- | --- | --- | --- | --- | --- |
|  | 1 | 2 | 3 | 4 | 5 | 6 |
| **7** | 8 | 9 | 10 | 11 | 12 | 13 |
| **14** | 15 | 16 | 17 | 18 | 19 | 20 |
| **21** | 22 | 23 | 24 | 25 | 26 | 27 |
| **28** | 29 | 30 | 31 |  |  |  |

**What do you think triggered your dizziness, vertigo, light-headedness?**

(Tick more than one if necessary)

| Sitting/standing up too quickly | [ ] | Hypoglycemia (low blood sugar levels) | [ ] |
| --- | --- | --- | --- |
| Looking up | [ ] | Medication(s) | [ ] |
| Quick head movement | [ ] | Stress / anxiety | [ ] |
| Turning over in bed | [ ] | Other- please specify: | [ ] |
| Dehydration | [ ] | …………………………………………… |  |
