# Supplementary material for: Reducing the burden of dizziness in middle-aged and older people: A multifactorial, tailored, single-blind randomized controlled trial
Source: PLoS Med. 2018 Jul 24;15(7):e1002620. doi: 10.1371/journal.pmed.1002620 (PMC6057644; doi:10.1371/journal.pmed.1002620)
Supplement: S4 Text — (DOCX) [file pmed.1002620.s004.docx]

**Reducing the burden of dizziness in middle aged and older people: a multifactorial, tailored, single blind, randomized controlled trial**

**Statistical Analysis Plan**

Jasmine C. Menant, Americo A. Migliaccio, Daina L Sturnieks, Cameron Hicks, Joanne Lo, Mayna Ratanapongleka, Jessica Turner, Kim Delbaere, Nickolai Titov, Daniela Meinrath, Catherine McVeigh, Jacqueline CT Close, Stephen R. Lord

**Date:** 31/03/2011

# 1. INTRODUCTION

Dizziness symptoms are common among older people and associated with a cascade of debilitating symptoms, such as reduced quality of life, depression and falls. The multifactorial aetiology of dizziness is a major barrier to establishing a clear diagnosis and offering effective therapeutic interventions. Only a few multidisciplinary interventions of dizziness have been conducted to date, all of a pilot nature and none tailoring the intervention to the specific causes of dizziness. This randomized-controlled trial aims to evaluate the effects of a multidisciplinary dizziness assessment followed by a tailored multifaceted intervention on dizziness handicap and self-reported dizziness, balance and gait in people aged 50 years and over with dizziness symptoms.

Three hundred community-dwelling people aged 50 years and over, who report significant dizziness in the previous year but are not currently receiving treatment for it will be recruited. A team of geriatrician, vestibular neuroscientist, psychologist, study coordinator and baseline assessor, will hold case conferences fortnightly to discuss and recommend appropriate therapy(ies) for each participant, based on their multidisciplinary baseline assessments. Participants will then be randomized to receive a 6-month intervention or usual care. The tailored, multifaceted intervention will comprise one or more of: physiotherapist-led vestibular rehabilitation program; eight-week internet-based cognitive-behavioral therapy; six-month Otago home-based exercise program; and/or; medical management. Participants will be followed for a 6-month period. Primary outcome measures will be dizziness-related quality of life measured with the Dizziness Handicap Inventory score, frequency of dizziness episodes recorded with monthly calendars over the six-month follow-up, choice stepping reaction time and step time variability. Secondary outcomes will include measures of, composite physiological falls risk, balance, anxiety, depression, neuroticism, fear of falling and orthostatic hypotension. Adverse effects will be monitored.

The study was approved by University of New South Wales Human Research Ethics Committee (HC12152). The study is registered with the Australian New Zealand Clinical Trials Registry ACTRN12612000379819. The findings will be disseminated in peer-reviewed journals and through professional and scientific conferences.

The protocol paper has been published:

**Menant JC, Migliaccio AA, Hicks C, Lo J, Meinrath D, Ratanapongleka M, Turner J, Sturnieks DL, Delbaere K, Titov N, McVeigh C, Close JC, Lord SR**. Tailored multifactorial intervention to improve dizziness symptoms and quality of life, balance and gait in dizziness sufferers aged over 50 years: protocol for a randomised controlled trial. BMC Geriatr. 2017 Feb 15;17(1):56.

# 2. DATASET ANALYSED

The main (ITT) dataset analysed will be constituted of all patients randomized onto the study irrespective of their allocations (intervention or control).

# 3. STUDY OUTCOMES

## Primary Outcomes

There are four primary outcomes; two measuring the impact of the dizziness symptoms and two concerned assessing balance and gait function.

1) The Dizziness Handicap Inventory (DHI) is a 25-item scale that assesses an individual’s perception of handicap due to dizziness and encompasses emotional, functional and physical burden. Scores of 0-30 represent mild symptoms, 31-60 moderate symptoms, and 61-100 severe symptoms (1). The total DHI score will be computed.

2) The total number of dizziness episodes during the six-month follow up will be monitored with monthly dizziness diaries and follow-up telephone calls as required.

3) Balance will be assessed using the choice-stepping reaction time (milliseconds), a validated measure of fall risk that also incorporates strength and reaction time (2).

4) Walking stability will be assessed by the mean step-time variability (coefficient of variation of step time; s), recorded as participants performed three walking trials at self-selected speed along on a 4-m long electronic mat placed in the middle of an 8-m long walkway (3, 4)

The four primary endpoints are:

1. The between-group difference in DHI (assessed using the total DHI score) at 6 months.
2. The between-group difference in dizziness episodes over the 6-month intervention period.
3. The between-group difference in the choice-stepping reaction time at 6 months.
4. The between-group difference in step time variability at 6 months.

## Secondary Outcomes

Secondary outcome measures will assess cardiovascular function, psychological function, fall risk and balance. The endpoints are a between-group difference in the following:

**Cardiovascular function**

1. Orthostatic hypotension: Tilt Table test - a decrease in systolic blood pressure ≥20 mmHg or a decrease of systolic blood pressure to ≤90 mmHg after 3min of upright standing, defines orthostatic hypotension whether or not symptoms occur (5)

**Psychological function**

1. Anxiety using the Generalized Anxiety Disorder 7 items scale (6)
2. Depression using the Patient Health Questionnaire -9 scale (7)
3. Neuroticism scale of the NEO-Five Factor Inventory (8)
4. Fear of falling using the Falls Efficacy Scale –International (9)

**Fall risk and balance**

1. Physiological Profile Assessment (PPA) summary score (10, 11)
2. Coordinated stability (12)

##

## Additional Measures in the intervention group

**Adherence** to all interventions documented with therapist records (vestibular rehabilitation / exercise program/ cognitive-behavioural therapy) and/or participant diaries (exercise program). The exercise physiologist responsible for the exercise intervention will estimate a global level of adherence (in five categories: 0%, <25%, 25–49%, 50–74% and ≥75%) during the 6-month intervention.

**Adverse events**: For the purpose of this trial, a serious adverse event is defined as an unwanted and usually harmful outcome (e.g., fall, seizure, cardiac event). A minor adverse event will be defined as musculoskeletal soreness that interferes with activities of daily living for more for 48 hours or requires medical attention. Adverse events will be monitored in the intervention group and not in the control group (for example, a fall during an exercise session) will be monitored with monthly calendars and telephone calls as required.

# 4. STATISTICAL ANALYSIS

## 4.1 Analysis principles

• Data will be coded to permit blinding to group allocation in the primary analysis.

• The primary analysis will be conducted by the in-house statistician, who has not been involved in any aspects of the data collection and entry.

• The primary analyses will be conducted in accordance with the intention-to-treat principle (analysis of all available data in the groups to which participants were allocated) (13)

• All tests are two-sided and the nominal level of α will be 5%.

• All statistical analyses will be unadjusted except where indicated.

• Subgroup analyses will be carried out irrespective of whether there is a significant treatment effect on the primary outcome.

• Where data are missing, we will report the number of observations; we will not impute missing values for the primary analyses. Sensitivity analysis will be conducted for the primary outcomes using multiple imputation of missing data.

• P-values will not be adjusted for multiplicity. However, the outcomes are clearly categorised by degree of importance (primary and secondary) and a limited number of subgroup analyses will be pre-specified.

• Descriptive statistics will include numbers and percentages of participants with specific scores or means and standard deviations in each group at the6-month follow-up.

• SPSS software will be used for analysis.

## 4.2 Dizziness handicap inventory, choice-stepping reaction time, step time variability (primary outcome)

To assess dizziness-related quality of life , the total score of the dizziness handicap inventory will be will be computed and treated as a continuous variable . The mean total choice-stepping reaction time computed from 24 trials and the mean step time variability computed from 3 walking trials will be analysed. The effect of group allocation on the outcomes at 6-month follow-up will be analysed using generalised linear models with baseline scores entered as covariates.

We do not foresee that any participant would be unable to perform any of the two primary outcome physical tests.

## 4.3 Frequency of dizziness episodes (primary outcome)

The number of dizziness episodes experienced during the 6-month follow-up will be analysed using negative binomial regression in SPSS, controlling for the length of follow-up (days) to estimate the difference in dizziness episodes frequency between the two groups. The relative risk and its 95% CI will be reported.

If outliers are present in the data or the model assumptions are grossly violated after treatment is included in the model, some sensitivity analyses will be conducted.

## 4.4 Secondary Outcomes

For the continuous secondary outcomes (detailed above), the effect of group allocation on continuously scored outcome measures at the 6-month follow-up will be analysed using generalized linear models with baseline scores entered into the models as covariates.

For the dichotomised secondary outcomes of orthostatic hypotension, logistic regression models will be used to compare the proportion of participants in each group, with baseline score as a covariate. Odds ratios and their 95% CIs will be reported.

## 4.5 Secondary Analyses

*Subgroup analyses primary and secondary outcome measures*

All subgroups will be defined by data collected prior to randomization.

Subgroup analyses will be conducted to determine intervention-specific effects on all primary outcomes and relevant secondary outcomes as outlined below:

- vestibular rehabilitation (intervention versus control among all participants recommended during the case-conferences to undertake this specific therapy) : coordinated stability and anxiety.
- home exercise program (intervention versus control among all participants recommended during the case-conferences to undertake this specific therapy): falls risk, coordinated stability, depression, fear of falling.
- cognitive-behavioural therapy (intervention versus control among all participants recommended during the case-conferences to undertake this specific therapy): anxiety, depression, neuroticism and fear of falling.
- medical management (intervention versus control among all participants recommended during the case-conferences to undertake this specific therapy): falls risk and orthostatic hypotension.

The subgroup analyses will be conducted in the same way as the main analysis. Unadjusted *p*-values will be reported.

## 4.6 Adherence data and adverse events

Adherence data and adverse events reported in the intervention group will be summarised.

##

## References

1. Jacobson GP, Newman CW. The development of the Dizziness Handicap Inventory. Archives of otolaryngology--head & neck surgery. 1990;116(4):424-7.

2. Lord SR, Fitzpatrick RC. Choice stepping reaction time: a composite measure of falls risk in older people. J Gerontol A Biol Sci Med Sci. 2001;56(10):M627-32.

3. Callisaya ML, Blizzard L, Schmidt MD, McGinley JL, Srikanth VK. Ageing and gait variability--a population-based study of older people. Age Ageing. 2010;39(2):191-7.

4. Hausdorff JM, Rios DA, Edelberg HK. Gait variability and fall risk in community-living older adults: a 1-year prospective study. Arch Phys Med Rehabil. 2001;82(8):1050-6.

5. Kenny RA, Ingram A, Bayliss J, Sutton R. Head-up tilt: a useful test for investigating unexplained syncope. Lancet. 1986;1(8494):1352-5.

6. Spitzer RL, Kroenke K, Williams JB, Lowe B. A brief measure for assessing generalized anxiety disorder: the GAD-7. Arch Intern Med. 2006;166(10):1092-7.

7. Spitzer RL, Kroenke K, Williams JB. Validation and utility of a self-report version of PRIME-MD: the PHQ primary care study. Primary Care Evaluation of Mental Disorders. Patient Health Questionnaire. Jama. 1999;282(18):1737-44.

8. Costa PT, McCrae, R.R. . NEO PI-R Professional Manual. Revised NEO Personality Inventory (NEO PI-R) and NEO Five-Factor Inventory (NEO-FFI). Odessa: Psychological Assessment Resources Inc.; 1992.

9. Delbaere K, Close JC, Mikolaizak AS, Sachdev PS, Brodaty H, Lord SR. The Falls Efficacy Scale International (FES-I). A comprehensive longitudinal validation study. Age Ageing. 2010;39(2):210-6.

10. Lord SR, Menz HB, Tiedemann A. A physiological profile approach to falls risk assessment and prevention. Phys Ther. 2003;83(3):237-52.

11. Lord SR, Ward JA, Williams P, Anstey KJ. Physiological factors associated with falls in older community-dwelling women. J Am Geriatr Soc. 1994;42(10):1110-7.

12. Lord SR, Ward JA, Williams P. Exercise effect on dynamic stability in older women: a randomized controlled trial. Arch Phys Med Rehabil. 1996;77(3):232-6.

13. White IR, Horton NJ, Carpenter J, Pocock SJ. Strategy for intention to treat analysis in randomised trials with missing outcome data. BMJ. 2011;342:d40.
