# Supplementary material for: Reducing the burden of dizziness in middle-aged and older people: A multifactorial, tailored, single-blind randomized controlled trial
Source: PLoS Med. 2018 Jul 24;15(7):e1002620. doi: 10.1371/journal.pmed.1002620 (PMC6057644; doi:10.1371/journal.pmed.1002620)
Supplement: S5 Text — (DOCX) [file pmed.1002620.s005.docx]

**Sensitivity analysis: primary outcome measures with imputed data**

We conducted multiple imputation for the dizziness handicap inventory (DHI) (n=31 missing), choice-stepping reaction time (n=48 missing) and step time variability (n=69 missing) data using IBM SPSS version 24. The monotone method with five iterations was used to impute DHI scores at re-assessments using baseline DHI scores as the predictor. The fully conditional specification method (iterative Markov chain Monte Carlo method) with 10 iterations was used to impute choice-stepping reaction times and step time variability data at re-assessment, using the baseline choice-stepping reaction time data and the baseline step time variability data, respectively.

Our sensitivity analysis, using generalized linear models showed similar results to the initial analyses as displayed in the table below. In brief, compared with the control group and controlling for baseline scores, DHI scores were still significantly lower in the intervention group versus the control group. No significant effects of the intervention were found for the choice-stepping reaction time or the step time variability.

**Table A. Mean (95% CI) between-group differences in primary outcome measures without and with imputed data**

|  | **Difference between groups (baseline adjusted) – available data** | **Difference between groups (baseline adjusted) – available plus imputed data** |
| --- | --- | --- |
|  | **Mean (95% CI), p** | **Mean (95% CI), p** |
| **Dizziness handicap inventory, score** | -3.7 (-6.2 to -1.2), p=0.003 | -3.5 (-5.9 to -1.08), p=0.004 |
| **Choice-stepping reaction time, milliseconds** | -15 (-40 to 10), p=0.246 | -15 (-38 to 8), p=0.195 |
| **Step time variability, s** | -0.001 (-0.002 to 0.001), p=0.497 | 0.001 (-0.001 to 0.002), p=0.435 |
