# Supplementary material for: Reducing the burden of dizziness in middle-aged and older people: A multifactorial, tailored, single-blind randomized controlled trial
Source: PLoS Med. 2018 Jul 24;15(7):e1002620. doi: 10.1371/journal.pmed.1002620 (PMC6057644; doi:10.1371/journal.pmed.1002620)
Supplement: S1 Table — (DOCX) [file pmed.1002620.s006.docx]

Table S1. Adverse events recorded during the study

| **Participant** | **Event** |
| --- | --- |
| 84 year-old female with a history of unexplained faints | Faint between tests at baseline assessment. |
| 61 year-old male with a history of similar fainting symptoms in the previous year | Faint during the tilt-table test of orthostatic hypotension. |
