# Supplementary material for: Reducing the burden of dizziness in middle-aged and older people: A multifactorial, tailored, single-blind randomized controlled trial
Source: PLoS Med. 2018 Jul 24;15(7):e1002620. doi: 10.1371/journal.pmed.1002620 (PMC6057644; doi:10.1371/journal.pmed.1002620)
Supplement: S2 Table — (DOCX) [file pmed.1002620.s007.docx]

**Table S2. Primary and relevant secondary outcome measures for the intervention and control participants eligible for vestibular rehabilitation, at baseline and follow-up assessments**

|  | **Baseline** | | **Follow-up** | | **Mean (95% CI) difference between groups at follow-up (baseline adjusted) or relative risk (RR) (95%CI)** |
| --- | --- | --- | --- | --- | --- |
|  | **Control (n=67)** | **Intervention**  **(n=54)** | **Control**  **(n=64)** | **Intervention**  **(n=50)** |  |
| **Primary outcome measures** | | | | | |
| DHI, mean (SD), score | 26.4 (17.4) | 28.7 (18.7) | 25.9 (16.6) | 21.2 (17.1) | **-6.3 (-10.2 to -2.3), p=0.002** |
| Dizziness frequency, median (IQR), (total number over 6 months) |  | | 40 (16 to 140) | 49 (12 to 119) | 0.98 (0.64 to 1.50), p=0.936 |
| Follow-up length, median (IQR), days |  | | 199 (188 to 211) | 205 (194 to 219) | Entered as covariate in above analysis |
| Choice stepping reaction time, median (IQR), milliseconds | 1025 (958 to 1160) | 1067 (961 to 1154) | 1033 (949 to 1148) | 1007 (939 to 1080) |  |
| Choice stepping reaction time, mean (SD), milliseconds | 1057 (190) | 1079 (137) | 1053 (156) | 1004 (124) | **-43 (-84 to -2), p=0.040** |
| Step time variability, median (IQR), s | 0.011 (0.009 to 0.016) | 0.013 (0.010 to 0.016) | 0.012 (0.008 to 0.017) | 0.013 (0.010 to 0.015) |  |
| Step time variability, mean (SD), s | 0.014 (0.009) | 0.015 (0.007) | 0.015 (0.011) | 0.015 (0.007) | -0.001 (-0.005 to 0.003), p=0.601 |
| **Secondary outcome measures** | | | | | |
| Coordinated stability, median (IQR), score | 2.0 (0.0 to 9.5) | 1.0 (0.0 to 10.0) | 2.0 (0.0 to 7.0) | 2.0 (0.0 to -10.0) |  |
| Coordinated stability, mean (SD), score | 4.2 (5.9) | 5.1 (6.8) | 4.6 (7.2) | 4.6 (5.8) | -0.6 (-2.5 to 1.4), p=0.570 |
| GAD-7, median (IQR), score | 1.0 (0.0 to 3.0) | 2.0 (0.0 to 5.0) | 1.0 (0.0 to 4.0) | 2.0 (0.0 to 3.0) |  |
| GAD-7, mean (SD), score | 2.3 (2.9) | 3.7 (4.6) | 2.7 (3.7) | 2.6 (3.1) | -0.6 (-1.6 to 0.5), p=0.296 |

DHI = Dizziness handicap inventory; GAD-7 = Generalized Anxiety Disorder 7 Item Scale. ^$^ Generalized linear models for continuous variables, negative binomial regression for dizziness frequency.
