# Supplementary material for: Reducing the burden of dizziness in middle-aged and older people: A multifactorial, tailored, single-blind randomized controlled trial
Source: PLoS Med. 2018 Jul 24;15(7):e1002620. doi: 10.1371/journal.pmed.1002620 (PMC6057644; doi:10.1371/journal.pmed.1002620)
Supplement: S3 Table — (DOCX) [file pmed.1002620.s008.docx]

**Table S3. Primary and relevant secondary outcome measures for the intervention and control participants eligible for the Otago home exercise program, at baseline and follow-up assessments**

|  | **Baseline** | | **Follow-up** | | **Mean (95% CI) difference between groups at follow-up (baseline adjusted) or relative risk (RR) (95%CI)** |
| --- | --- | --- | --- | --- | --- |
|  | **Control (n=39)** | **Intervention**  **(n=37)** | **Control**  **(n=37)** | **Intervention**  **(n=32)** |  |
| **Primary outcome measures** | | | | | |
| DHI, mean (SD), score | 28.6 (19.1) | 38.4 (21.7) | 28.0 (17.3) | 34.9 (20.0) | -1.1 (-5.3 to 3.0), p=0.593 |
| Dizziness frequency, median (IQR), (total number over 6 months) |  | | 92 (17 to 178) | 112 (41 to 200) | 1.06 (0.67 to 1.67), p=0.818 |
| Follow-up length, median (IQR), days |  | | 194 (177 to 206) | 203 (192 to 227) | Entered as covariate in above analysis |
| Choice stepping reaction time, median (IQR), milliseconds | 1171 (990 to 1326) | 1126 (986 to 1317) | 1136 (1011 to 1262) | 1077 (1000 to 1178) |  |
| Choice stepping reaction time, mean (SD), milliseconds | 1165 (184) | 1187 (260) | 1141 (154) | 1120 (191) | -22 (-79 to 35), p=0.441 |
| Step time variability, median (IQR), s | 0.017 (0.012 to 0.023) | 0.017 (0.013 to 0.025) | 0.017 (0.012 to 0.024) | 0.014 (0.013 to 0.028) |  |
| Step time variability, mean (SD), s | 0.020 (0.011) | 0.021 (0.012) | 0.020 (0.013) | 0.021 (0.013) | -0.002 (-0.007 to 0.004) , p=0.565 |
| **Secondary outcome measures** | | | | | |
| PPA, mean (SD), score | 1.67 (0.70) | 1.78 (0.73) | 1.84 (0.73) | 1.46 (0.53) | **-0.37 (-0.68 to -0.06), p=0.018** |
| Coordinated stability, median (IQR), score | 12.5 (3.5 to 23.3) | 11.0 (4.5 to 24.5) | 5.5 (2.5 to 18.0) | 11.0 (3.0 to 24.0) |  |
| Coordinated stability, mean (SD), score | 14.2 (12.1) | 13.8 (11.8) | 10.9 (12.1) | 14.6 (12.9) | 2.5 (-0.6 to 5.5), p=0.116 |
| PHQ-9, median (IQR), score | 3.0 (2.0 to 6.0) | 4.0 (2.0 to 7.5) | 3.0 (2.0 to 4.5) | 3.0 (1.3 to 9.8) |  |
| PHQ-9, mean (SD), score | 5.3 (5.9) | 5.4 (4.9) | 3.9 (3.7) | 5.9 (6.4) | 1.1 (-0.2 to 2.4), p=0.107 |
| Icon-FES, median (IQR), score | 21.0 (16.0 to 28.0) | 23.5 (21.0 to 27.0) | 21.0 (15.0 to 27.3) | 22.0 (17.0 to 27.0) |  |
| Icon-FES, mean (SD), score | 22.2 (6.7) | 24.2 (8.2) | 21.4 (6.6) | 22.6 (7.3) | -0.6 (-3.6 to 2.4), p=0.716 |

DHI = Dizziness handicap inventory, PPA= Physiological Profile Assessment; PHQ-9 = Patient Health Questionnaire 9 Item Scale; Icon-FES = Iconographical Falls Efficacy Scale. ^$^Generalized linear models for continuous variables, negative binomial regression for dizziness frequency.
