# Supplementary material for: Reducing the burden of dizziness in middle-aged and older people: A multifactorial, tailored, single-blind randomized controlled trial
Source: PLoS Med. 2018 Jul 24;15(7):e1002620. doi: 10.1371/journal.pmed.1002620 (PMC6057644; doi:10.1371/journal.pmed.1002620)
Supplement: S4 Table — CBT, cognitive-behavioural therapy. (DOCX) [file pmed.1002620.s009.docx]

**Table S4. Primary and relevant secondary outcome measures for the intervention and control participants eligible for the cognitive-behavioral therapy, at baseline and follow-up assessments**

|  | **Baseline** | | **Follow-up** | | **Mean (95% CI) difference between groups at follow-up (baseline adjusted) or relative risk (RR) (95%CI)** |
| --- | --- | --- | --- | --- | --- |
|  | **Control (n=22)** | **Intervention (n=29)** | **Control (n=17)** | **Intervention**  **(n=28)** |  |
| **Primary outcome measures** | | | | | |
| DHI, mean (SD), score | 33.5 (15.7) | 39.4 (23.4) | 34.2 (16.0) | 29.4 (22.1) | -**9.2 ( -16.7 to -1.8), p=0.015** |
| Dizziness frequency, median (IQR), (total number over 6 months) |  | | 142 (21 to 205) | 55 (17 to 153) | 0.60 (0.33-1.07), p=0.081 |
| Follow-up length, median (IQR), days |  | | 201 (189 to 237) | 197 (186 to 214) | Entered as covariate in above analysis |
| Choice stepping reaction time, median (IQR), milliseconds | 1023 (927 to 1208) | 1075 (965 to 1162) | 1004 (954 to 1190) | 1044 (969 to 1139) |  |
| Choice stepping reaction time, mean (SD), milliseconds | 1076 (188) | 1093 (207) | 1066 (160) | 1074 (196) | -10 (-70 to 51), p=0.754 |
| Step time variability, median (IQR), s | 0.013 (0.010 to 0.023) | 0.014 (0.011-0.017) | 0.013 (0.010 to 0.018) | 0.012 (0.009 to 0.019) |  |
| Step time variability, mean (SD), s | 0.016 (0.008) | 0.016 (0.010) | 0.014 (0.006) | 0.016 (0.010) | 0.000 (-0.003 to 0.003), p=0.830 |
| **Secondary outcome measures** | | | | | |
| GAD-7, median (IQR), score | 7.0 (5.0 to 10.3) | 8.0 (7.0 to 12.5) | 6.0 (2.5 to 11.0) | 3.5 (1.3 to 6.8) |  |
| GAD-7, mean (SD), score | 7.9 (4.2) | 9.5 (4.2) | 7.4 (5.7) | 5.0 (4.6) | **-3.6 (-6.6 to -0.7), p=0.015** |
| PHQ-9 ,median (IQR), score | 12.5 (8.8 to 14.5) | 9.0 (6.0 to 13.0) | 9.0 (4.5 to 15.0) | 5.5 (2.0 to 10.0) |  |
| PHQ-9, mean (SD), score | 12.4 (5.9) | 9.6 (4.8) | 10.6 (6.8) | 7.0 (6.5) | -1.0 (-3.8 to 1.8), p=0.487 |
| Neuroticism, mean (SD), score | 26.3 (9.2) | 26.4 (6.7) | 24.7 (6.9) | 24.9 (7.0) | 0.9 (-2.0 to 3.7), p=0.544 |
| Icon-FES, median (IQR), score | 19.0 (14.8 to 25) | 21.5 (15.0 to 27.0) | 20.0 (15.5 to 27.0) | 18.0 (13.5 to 24.50) |  |
| Icon-FES, mean (SD), score | 20.0 (6.6) | 22.4 (9.2) | 21.6 (7.0) | 20.8 (8.4) | -2.7 (-6.3 to 1.2), p=0.172 |

DHI = Dizziness handicap inventory; GAD-7 = Generalized Anxiety Disorder 7 Item Scale; PHQ-9 = Patient Health Questionnaire 9 Item Scale; Icon-FES = Iconographical Falls Efficacy Scale. ^$^Generalized linear models for continuous variables, negative binomial regression for dizziness frequency
