# Supplementary material for: Reducing the burden of dizziness in middle-aged and older people: A multifactorial, tailored, single-blind randomized controlled trial
Source: PLoS Med. 2018 Jul 24;15(7):e1002620. doi: 10.1371/journal.pmed.1002620 (PMC6057644; doi:10.1371/journal.pmed.1002620)
Supplement: S5 Table — (DOCX) [file pmed.1002620.s010.docx]

**Table S5. Primary and relevant secondary outcome measures for the intervention and control participants eligible for the medical management (letter to General Practitioner, Falls Clinic), at baseline and follow-up assessments**

|  | **Baseline** | | **Follow-up** | | **Mean (95% CI) difference between groups at follow-up (baseline adjusted) or relative risk (RR) (95%CI)** |
| --- | --- | --- | --- | --- | --- |
|  | **Control (n=55)** | **Intervention (n=62)** | **Control (n=49)** | **Intervention (n=57)** |  |
| **Primary outcome measures** | | | | | |
| DHI, mean (SD), score | 22.9 (14.9) | 28.0 (19.0) | 22.9 (16.4) | 22.4 (17.5) | **-4.6 (-8.4 to -0.8), p=0.019** |
| Dizziness frequency, median (IQR), (total number over 6 months) |  | | 38 (13 to 149) | 41 (13 to 117) | 0.83 (0.53 to 1.29), p=0.403 |
| Follow-up length, median (IQR), days |  | | 197 (188 to 240) | 203 (185 to 232) | Entered as covariate in above analysis |
| Choice stepping reaction time, median (IQR), milliseconds | 1040 (946 to 1155) | 1076 (961 to 1174) | 1040 (961 to 1124) | 1043 (946 to 1137) | -25 (-60 to 9), p=0.147 |
| Choice stepping reaction time, mean (SD), milliseconds |  |  |  |  |  |
| Step time variability, median (IQR), s | 0.013 (0.010 to 0.020) | 0.013 (0.010 to 0.019) | 0.012 (0.009 to 0.016) | 0.013 (0.011 to 0.017) | -0.001 (-0.002 to 0.004), p=0.555 |
| Step time variability, mean (SD), s |  |  |  |  |  |
| **Secondary outcomes measures** | | | | | |
| PPA, mean (SD), score | 0.94 (0.88) | 0.99 (0.89) | 1.20 (0.80) | 1.01 (0.71) | -0.21 (-0.43 to 0.02), p=0.068 |
| Orthostatic hypotension, No (%) | 25 (46) | 30 (48) | 19 (40) | 21 (37) |  |

DHI = Dizziness handicap inventory; PPA= Physiological Profile Assessment. ^$^Generalized linear models for continuous variables, negative binomial regression for dizziness frequency
